# Overexpression of Lotus *NnSWEET4a* Alters Sugar Homeostasis and Induces Salt Hypersensitivity in *Arabidopsis*

**DOI:** 10.3390/plants15162542

**Published:** 2026-08-21

**Authors:** Shilong Zhao, Xiangxin Lu, Zongyue Li, Xiaoyi Zhang, Siying Chen, Yan Gao, Jiashi Peng, Tianyu Gu

**Affiliations:** 1 Hunan Key Laboratory of Economic Crops Genetic Improvement and Integrated Utilization, Hunan University of Science and Technology, Xiangtan 411201, China; zhaoshilong@mail.hnust.edu.cn (S.Z.); luxiangx0221@163.com (X.L.); lzy_epsilon@163.com (Z.L.); xiaoyizhang2026@163.com (X.Z.); swing9510c@163.com (S.C.); blessedgy@hnust.edu.cn (Y.G.); jspeng@hnust.edu.cn (J.P.); 2 Hunan Provincial Engineering Research Center for the Deep Processing and Health Benefits of Xianglian, School of Life and Health Sciences, Hunan University of Science and Technology, Xiangtan 411201, China

**Keywords:** *Nelumbo nucifera*, SWEET, sugar, SOS, salt stress

## Abstract

SWEET (Sugars Will Eventually be Exported Transporter) proteins constitute a conserved family of sugar transporters that play pivotal roles in carbohydrate allocation and stress responses. In this study, we systematically identified 14 SWEET homologs in the genome of sacred lotus (*Nelumbo nucifera*) and validated their transport activity for both hexoses and sucrose. Subsequent analysis revealed that stress-responsive elements are the most enriched promoter sequences of *NnSWEET* genes. Quantitative expression profiling of the members found that *NnSWEET4a* was strongly upregulated under salt stress. NnSWEET4a was localized at the plasma membrane; its expression conferred salt sensitivity in both yeast and *Arabidopsis thaliana*. Transgenic Arabidopsis lines overexpressing *NnSWEET4a* exhibited substantial downregulation of the *SOS3*–*SOS2*–*SOS1* signaling module and concomitant alterations in cellular sugar homeostasis. Further analysis revealed that exogenous sugar application aggravated salt sensitivity and SOS pathway inhibition in *NnSWEET4a* transgenic plants, and *NnSWEET15*-overexpressing Arabidopsis recapitulated identical phenotypic and molecular responses, including salt sensitivity and repression of *SOS* genes. These findings indicate that NnSWEET4a impairs salt tolerance through disruption of sugar homeostasis. The results establish a mechanistic framework for future investigations into SWEET-dependent regulation of sugar homeostasis and salt stress adaptation.

## 1. Introduction

Salt stress represents one of the major environmental constraints limiting plant growth and development and agricultural productivity worldwide [1,2]. Excessive accumulation of sodium (Na^+^) in plant cells disrupts cellular ion homeostasis and concurrently induces osmotic stress and oxidative damage [3,4]. Plants have evolved a series of sophisticated mechanisms for coping with over-accumulated Na^+^, including extruding Na^+^ out of cells, restricting Na^+^ transport from the root to shoot, sequestering Na^+^ in vacuoles, activating antioxidant enzymes and balancing osmotic pressure via synthesizing compatible osmolytes such as soluble sugar [5,6]. Sugars are not merely inert osmolytes but a regulatory factor governing plant survival under salt stress. Accumulating evidence indicates that sugars exert a dual-role effect in plant responses to salt stress [7]. Moderate sugar accumulation coupled with efficient carbon partitioning enhances osmotic adjustment and supports antioxidant system activity, thereby improving salt tolerance [8,9]. In contrast, perturbations in sugar homeostasis or aberrant sugar signaling compromise cellular resilience and render plants hypersensitive to salinity [10,11,12].

SWEET (Sugars Will Eventually be Exported Transporters) proteins represent a newly identified class of sugar transporters widely distributed across the plant kingdom. Structurally, plant SWEET proteins typically comprise seven transmembrane helices (TMs) and harbor two MtN3/saliva domains (Pfam ID: PF03083) [13,14,15]. Based on evolutionary relationships, members of the SWEET family are generally categorized into four clades. Clade I and Clade II primarily function as hexose (glucose and fructose) transporters [16,17]. Clade III preferentially transports sucrose [18,19], and Clade IV proteins are predominantly tonoplast-localized and implicated in fructose transport [20,21]. Recent studies have demonstrated that SWEET proteins not only play a critical role in sugar transport within plants but also participate in plant responses to salt stress. For instance, *DsSWEET17* from *Dianthus spiculifolius* and *OsSWEET13*/*OsSWEET15* from *Oryza sativa* conferred salt tolerance by maintaining intracellular sugar homeostasis [22,23], whereas *NtSWEET12i* in *Nicotiana tabacum* contributes to salinity acclimation by modulating ABA (Abscisic Acid) signaling, proline biosynthesis and ROS (Reactive Oxygen Species) scavenging [24]. *JcSWEET16* from *Jatropha carcass* and *HcSWEET4a* from *Hemerocallis citrina* exerted comparable functions in improving salt resistance [25,26]. In contrast, AtSWEET15 in *Arabidopsis thaliana* and MeSWEET15a/b from *Manihot esculenta* function as negative regulators. Overexpression of *AtSWEET15* impairs cell viability by modulating cell-membrane integrity, thereby increasing salt sensitivity and accelerating leaf senescence [12]. *MeSWEET15a/b*-silenced cassava plants accumulated sucrose in their leaves, improving their salt-stress tolerance by strengthening multiple defense strategies associated with cellular osmotic potential, redox homeostasis, and activation of the TOR (Target of Rapamycin) signaling pathway [27]. Collectively, SWEET family proteins may exert balanced dual functions in coordinating plant sugar metabolism and salt adaptation.

Lotus (*Nelumbo nucifera* Gaertn.) is an ancient, perennial, aquatic angiosperm belonging to the family Nelumbonaceae, which contains a single genus, Nelumbo, with two extant species: *N. nucifera* and *N. lutea* [28,29]. Lotus is popularly cultivated in Asia, and it is primarily categorized as a seed, rhizome and flower lotus based on agronomic traits and specific utilization purposes [30,31]. Currently, lotus cultivation is increasingly threatened by salt stress that induces chlorosis, wilting, photosynthetic inhibition, and severe reductions in biomass and rhizome quality, thereby constraining the sustainable expansion of the lotus industry [32,33]. Consequently, identifying and characterizing salinity-responsive genes represents a critical priority in the modern breeding of lotus. With the completion of whole-genome sequencing of *N. nucifera*, several genes from diverse families have been reported to participate in plant salt stress adaptation [34,35,36,37]. However, to date, no comprehensive identification or functional characterization of the *SWEET* gene family has been conducted in *N. nucifera*, and its potential involvement in salinity response remains entirely unexplored.

In this study, we systematically identified the *SWEET* gene family in *N. nucifera*, including sequence characteristics, phylogenetic relationships, genomic synteny, cis-acting element analysis and subcellular localization assays. Notably, *NnSWEET4a* was demonstrated to be involved in salt stress response, potentially acting through the SOS (Salt Overly Sensitive) signaling pathway in a sugar-dependent manner.

## 2. Results

### 2.1. Identification and Analysis of NnSWEET Family in Lotus

A total of 14 *NnSWEET*-encoding genes were identified in lotus via homology alignment and conserved domain validation. These genes were designated *NnSWEET1a*–*NnSWEET17* based on their sequence homology to SWEET members from the dicot *A. thaliana* and monocot *O. sativa.* All predicted NnSWEET proteins exhibit characteristic hydrophobicity, yet display notable variation in coding sequence length, protein stability, and subcellular localization (Table 1). Chromosomal mapping revealed that the 14 *NnSWEET* genes are non-uniformly distributed across chromosomes 1, 3, 4, and 5 of the genomes, with chromosome 3 harboring the largest cluster (seven genes), followed by chromosome 1 (four genes), chromosome 4 (two genes), and chromosome 5 (one gene) (Figure S1). Structural analysis confirmed that each NnSWEET protein contains three evolutionarily conserved motifs and two canonical MtN3/saliva domains, which underpin their core biological functions. Moreover, all *NnSWEET* genes share a highly conserved exon–intron architecture, comprising four to five introns within their coding sequences (Figure S2).

All *NnSWEET* genes were detectable in at least one tissue according to publicly available gene expression data [38]. Among them, *NnSWEET2* displayed constitutive expression across all examined tissues, whereas the remaining members exhibited distinct tissue-preferential expression patterns. Specifically, *NnSWEET1a*, *NnSWEET1b*, *NnSWEET2*, *NnSWEET4a*, and *NnSWEET15* showed markedly elevated expression in seeds and rhizomes; *NnSWEET8* and *NnSWEET10* were predominantly expressed in stamens; and *NnSWEET16* demonstrated preferential accumulation in leaves and receptacles (Figure S3a). qRT-PCR validation corroborated the RNA-seq expression profiles for *NnSWEET2*, *NnSWEET4a*, *NnSWEET15*, and *NnSWEET16*. In contrast, *NnSWEET10* exhibited divergent tissue-specific expression between the two datasets (Figure S3b–g), a discrepancy likely attributable to variables including plant developmental stage and cultivar differences. These widespread tissue expression patterns indicated potential involvement of *NnSWEET* genes in diverse developmental processes of lotus.

### 2.2. Phylogenetic and Collinearity Analysis of the NnSWEET Family

**Figure 1:**
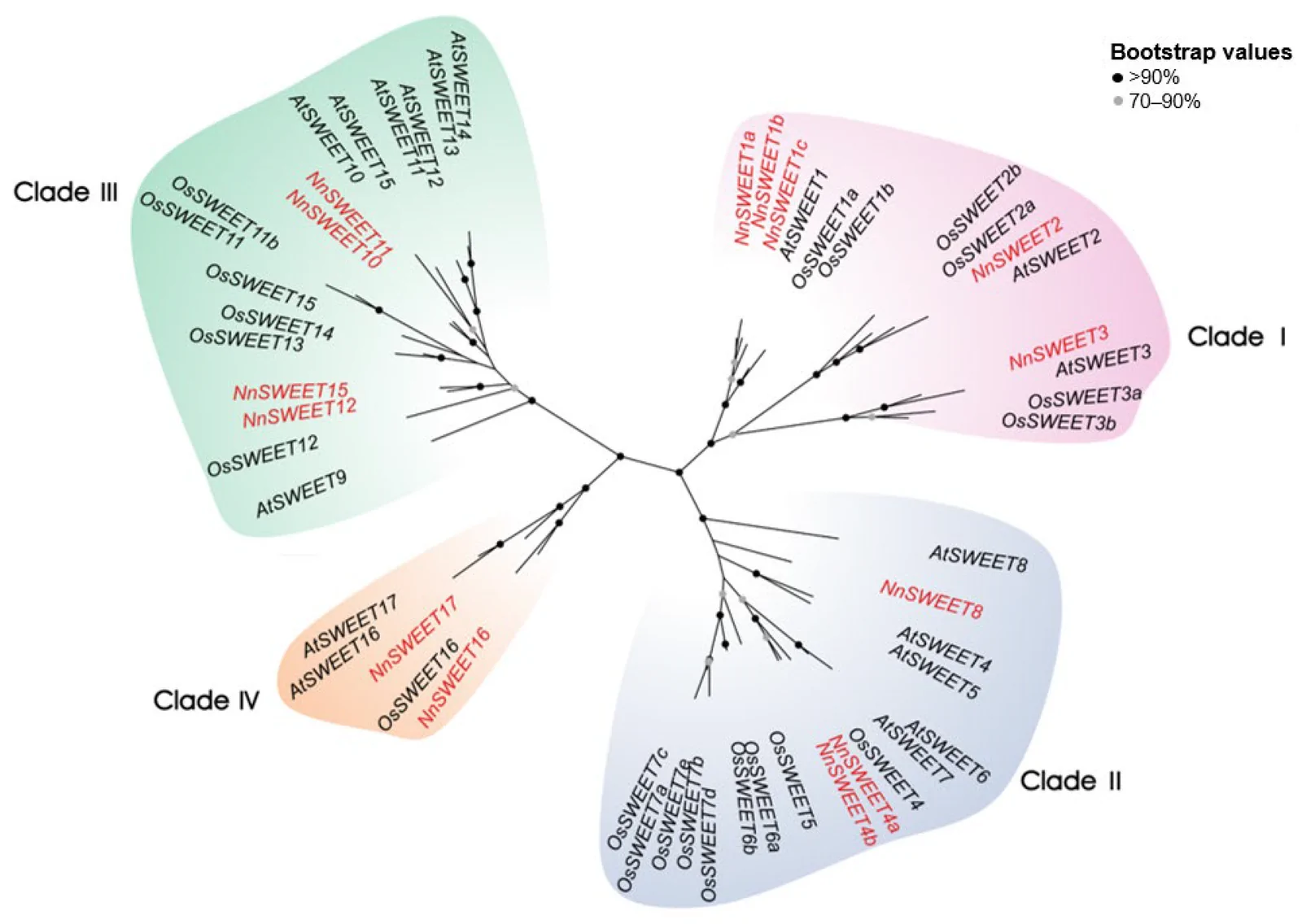
A phylogenetic tree of SWEETs from lotus (*N. nucifera*), Arabidopsis (*A. thaliana*) and rice (*O. sativa*) was constructed based on the neighbor-joining (NJ) method and 1000 bootstrap replications. Four subgroups were divided as Clades I–IV according to the evolutionary distance. NnSWEETs are highlighted in red. Black solid circles at nodes represent bootstrap support values > 90%, and gray solid circles indicate bootstrap values ranging from 70% to 90%. Branch lengths are proportional to sequence divergence.

In order to clarify evolutionary relationships, NnSWEETs were compared with their homologs in other plant species. Specifically, 17 AtSWEETs from *A. thaliana*, 22 OsSWEETs from *O. sativa* and 14 NnSWEET protein sequences were aligned to construct an unrooted phylogenetic tree. These SWEET proteins were clearly divided into four clades (I to IV), which were in accordance with previous phylogenetic classifications of SWEETs [17]. Among them, clade I contained the largest number of NnSWEETs (five members), while clade IV possessed the fewest NnSWEETs (NnSWEET16 and NnSWEET17), and clades II and III contained three and four NnSWEETs (Figure 1).

Gene duplication is a major mechanism driving gene family expansion. Five pairs of segmental duplication events were identified, *NnSWEET1a/1b*, *NnSWEET4a/4b*, *NnSWEET10/11*, *NnSWEET10/15* and *NnSWEET12/15,* on separate chromosomes in *N. nucifera*, whereas four pairs of segmental duplication events were identified in another species, *Nelumbo lutea* (Figure S4a,b). The synteny relationship of SWEET genes among *N. nucifera*, *N. lutea, A. thaliana* and *O. sativa* showed that a total of 20 pairs of orthologs were identified between *N. nucifera* and *N. lutea*, 10 pairs between *N. nucifera* and *A. thaliana,* and 11 pairs between *N. nucifera* and *O. sativa,* respectively. Furthermore, the non-synonymous (Ka)/synonymous (Ks) ratios of all the gene pairs were calculated to be less than one, suggesting that the genes had conservative function and underwent purifying selection (Figure S4c,d; Table S2).

### 2.3. Sugar Transport Activity of NnSWEETs

**Figure 2:**
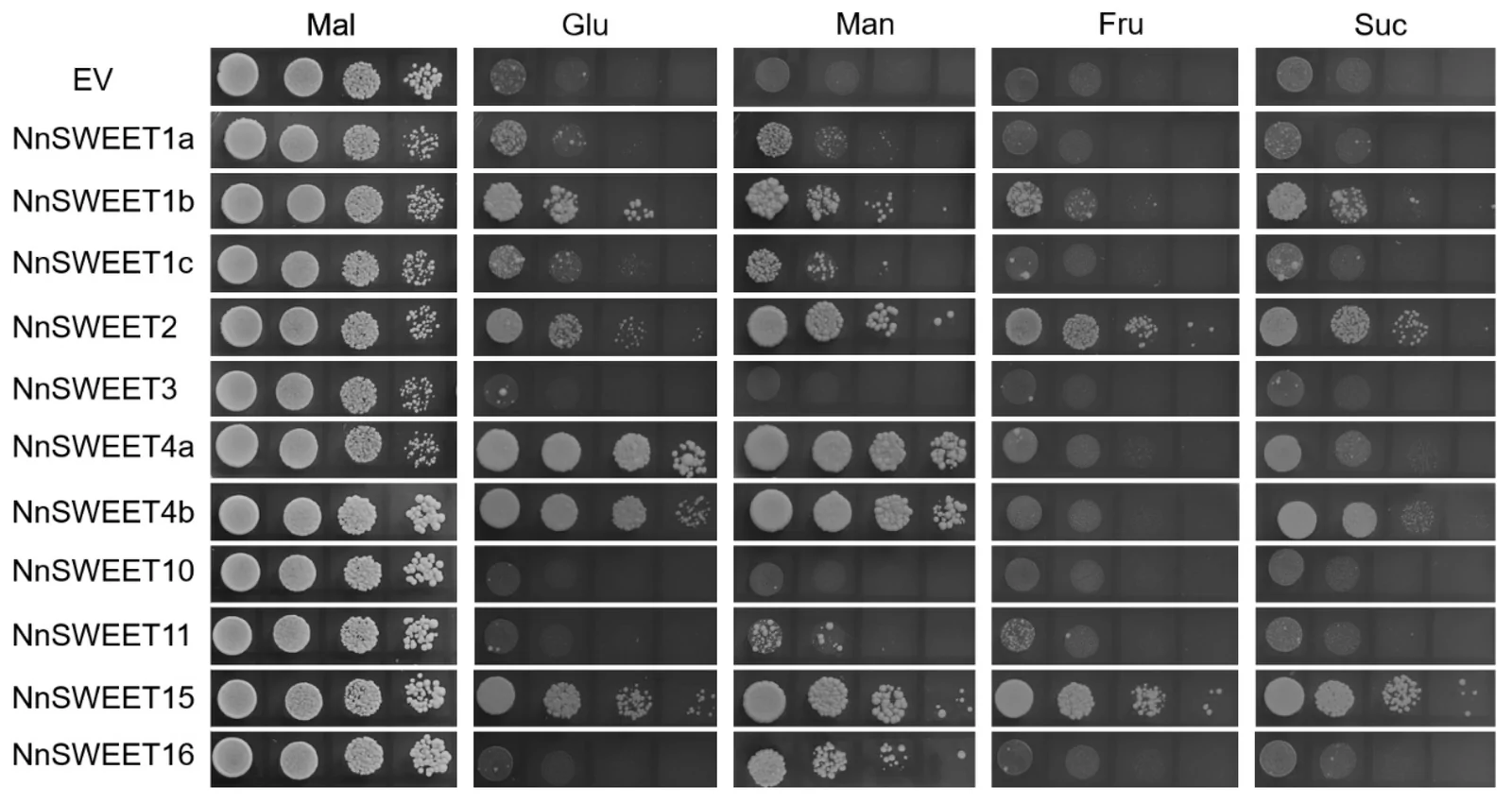
Transport activity of NnSWEETs in the yeast mutant *EBY.VW4000*. Yeast cells expressing an empty vector pDR196 (EV) or NnSWEETs cultured on solid synthetic deficient media without uracil (SD/-Ura) supplemented with 2% (*w*/*v*) maltose (Mal), 2% (*w*/*v*) glucose (Glu), 2% (*w*/*v*) mannose (Man), 2% (*w*/*v*) fructose (Fru) or 2% (*w*/*v*) sucrose (Suc) as the sole carbon source.

Given that SWEET family proteins generally possess sugar-transport activity, we then employed the hexose transporter-deficient yeast strain EBY.VW4000 [39] to characterize the sugar transport activity of NnSWEETs in lotus. After expressing 11 of the 14 cloned NnSWEETs in *EBY.VW4000*, we found that different members exhibited different substrate transport characteristics (Figure 2). NnSWEET1b, NnSWEET2, NnSWEET4a, NnSWEET4b and NnSWEET15 exhibited broad-spectrum transport activity, supporting yeast growth on media containing glucose, mannose, fructose, or sucrose as the sole carbon source. In contrast, yeast strains expressing *NnSWEET3* or *NnSWEET10* failed to grow on any of the tested sugars, even though qRT-PCR results confirmed that these two genes were successfully expressed in yeast (Figure S5). It is speculated that NnSWEET3 and NnSWEET10 may be located in the endomembrane system, where the yeast heterologous system is considered to be not suitable for the test of sugar transport capabilities of non-plasma membrane-localized SWEETs [16]. Their transport properties therefore remain to be determined using alternative approaches. The remaining NnSWEETs displayed distinct substrate preferences: NnSWEET1a and NnSWEET1b transported glucose and mannose; NnSWEET16 was specific to mannose and NnSWEET11 facilitated transport of mannose and fructose.

### 2.4. Salt Stress Induces Expression of NnSWEET4a

**Figure 3:**
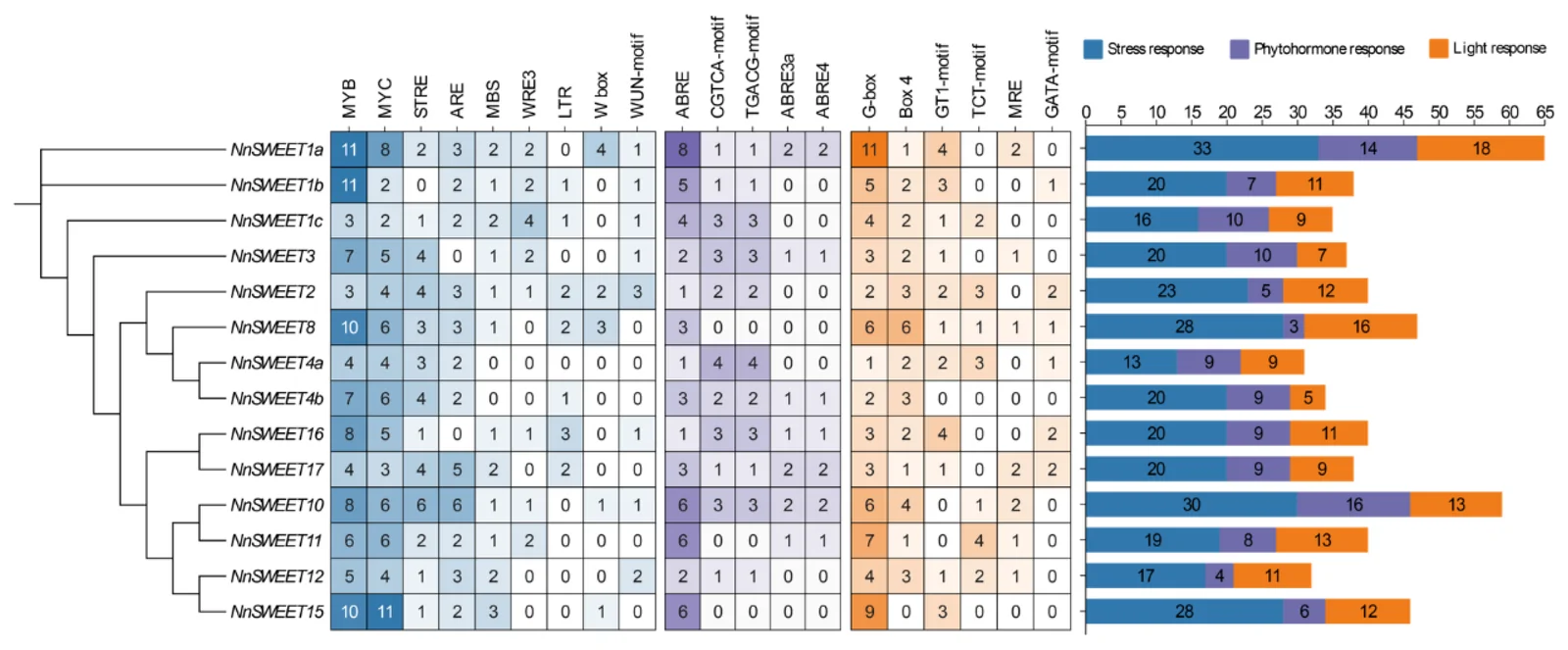
Statistical analysis of cis-regulatory elements in the 2 kb upstream promoter regions of *NnSWEET* genes. The numbers in each box indicate the count of cis-elements in *NnSWEETs*. The top 20 items ranked by total cis-element number were selected to construct the plot. The cell color depth corresponds to the number of cis-elements; darker color indicates a higher count of cis-elements. Blue for stress-response, purple for phytohormone-response, and orange for light-response.

To gain insight into the transcriptional regulation of *NnSWEET* genes and infer their potential physiological roles, we conducted a comprehensive cis-regulatory element analysis of the 2 kb upstream promoter regions. The number of predicted cis-elements varied substantially across the gene family, ranging from 31 elements in *NnSWEET4a* to 65 in *NnSWEET1* (Figure 3). Strikingly, stress-responsive elements were significantly enriched relative to hormone- or light-responsive elements across all promoters (Figure 3), implying broad transcriptional responsiveness of these genes to stresses. This enrichment supports a conserved role for *NnSWEETs* in stress adaptation.

**Figure 4:**
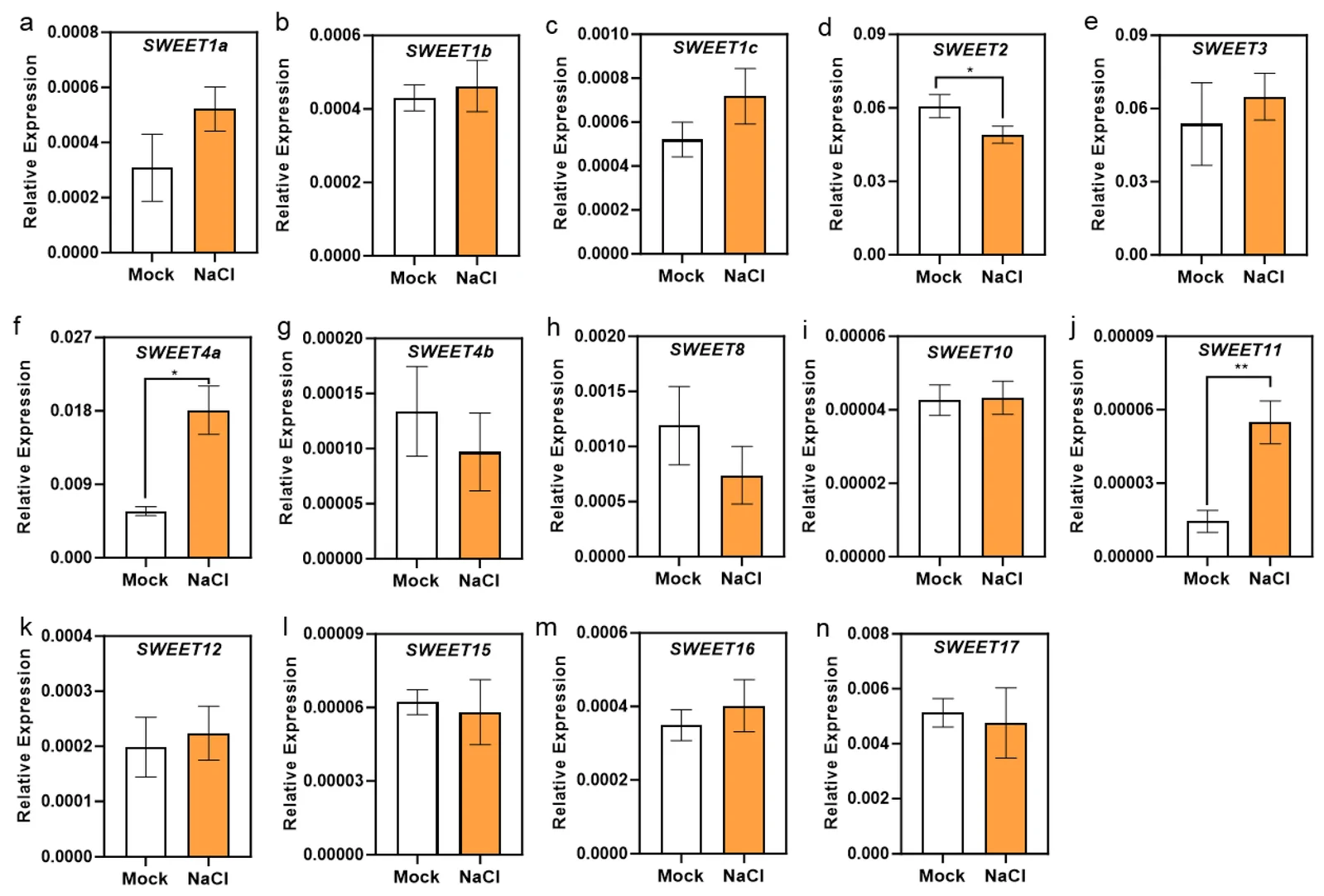
Expression of all 14 *NnSWEET* genes in lotus roots under 50 mM NaCl treatment for 24 h detected by qRT-PCR. (**a**) *SWEET1a*. (**b**) *SWEET1b*. (**c**) *SWEET1c*. (**d**) *SWEET2*. (**e**) *SWEET3*. (**f**) *SWEET4a*. (**g**) *SWEET4b*. (**h**) *SWEET8*. (**i**) *SWEET10*. (**j**) *SWEET11*. (**k**) *SWEET12*. (**l**) *SWEET15*. (**m**) *SWEET16*. (**n**) *SWEET17*. *NnActin* was used as an internal control. Data are mean ± SD; *n* = 3 for independent biological replicates; Student’s *t*-test: * *p* < 0.05 and ** *p* < 0.01.

Given that salinity is a major constraint on plant productivity, and prior evidence implicates certain SWEETs in salt tolerance modulation [12,22,23,24,25,26,27,40], we conducted qRT-PCR detection for all 14 *NnSWEET* genes under 50 mM NaCl treatment. Among them, *NnSWEET4a* and *NnSWEET11* exhibited robust and statistically significant upregulation (~three-fold increase 24 h post-treatment). Even so, *NnSWEET11* showed extremely low basal transcript abundance in lotus, reaching only 1% of the expression level of *NnSWEET4a*. Meanwhile, *NnSWEET2* expression declined modestly, and the remaining genes displayed no significant change (Figure 4).

### 2.5. NnSWEET4a Confers Salt Sensitivity in Yeast

**Figure 5:**
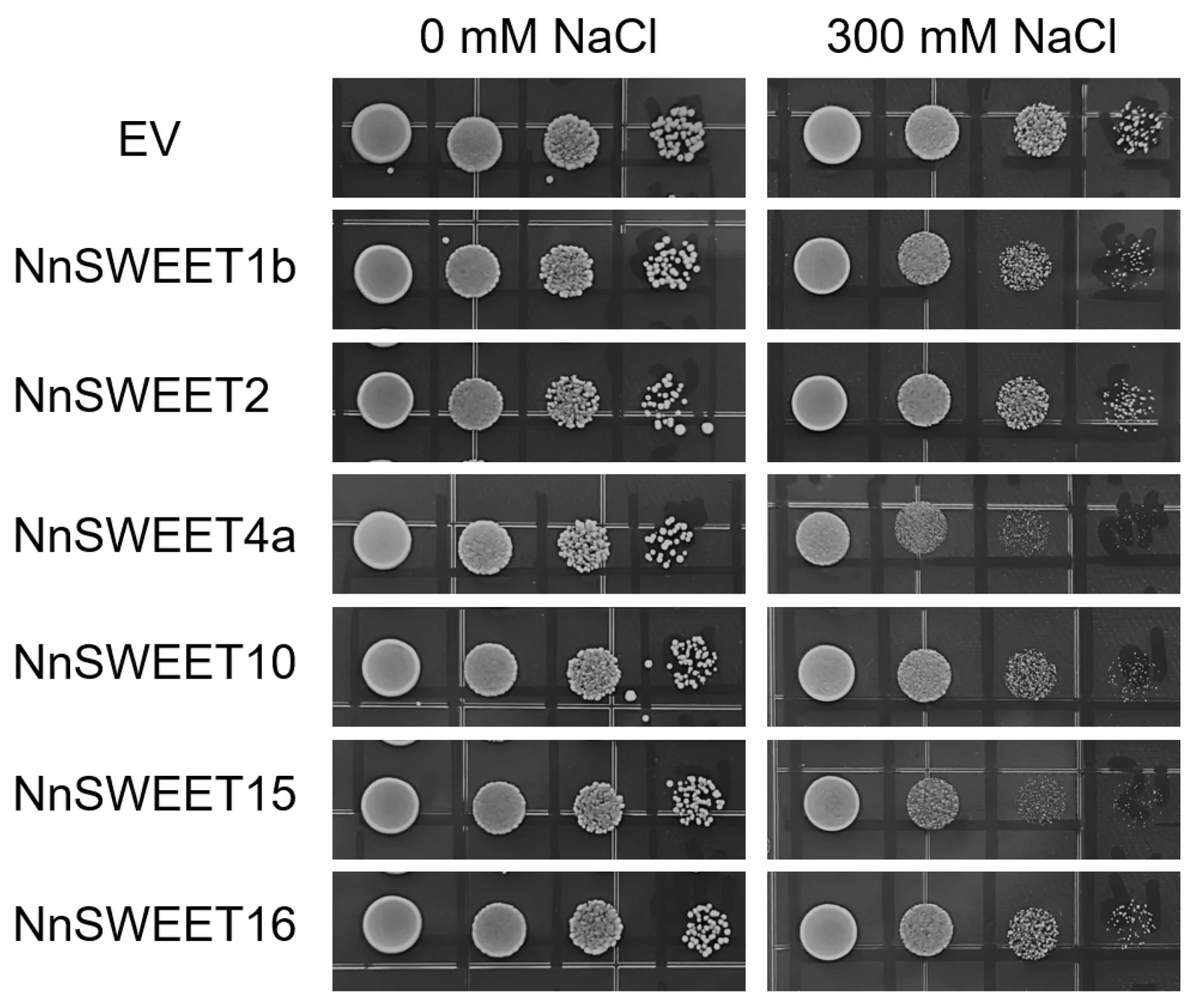
NnSWEETs confer sodium sensitivity to yeast. Yeast *EBY.VW4000* cells harboring the empty vector pDR196 (EV) or selected *NnSWEET* genes were grown on SD medium containing 2% (*w*/*v*) maltose with or without 300 mM NaCl.

To further assess the roles of *NnSWEETs* in cellular salt tolerance and take advantage of yeast as a robust eukaryotic heterologous expression system, we evaluated the growth performance of *EBY.VW4000* transformants expressing individual *NnSWEETs* under 300 mM NaCl. As expected, high salinity severely inhibited growth of all strains. Notably, strains expressing *NnSWEET4a* or *NnSWEET15* displayed significantly greater growth inhibition compared with the empty-vector control (Figure 5). In contrast, transformants expressing other *NnSWEETs* grew comparably to the EV control. The salt stress-induced expression pattern of *NnSWEET4a* and its functional role in modulating cellular sensitivity to salinity prompted its selection as the candidate in this story to further investigate the involvement of *NnSWEET* genes in salt tolerance mechanisms.

### 2.6. NnSWEET4a Was Localized on the Plasma Membrane

**Figure 6:**
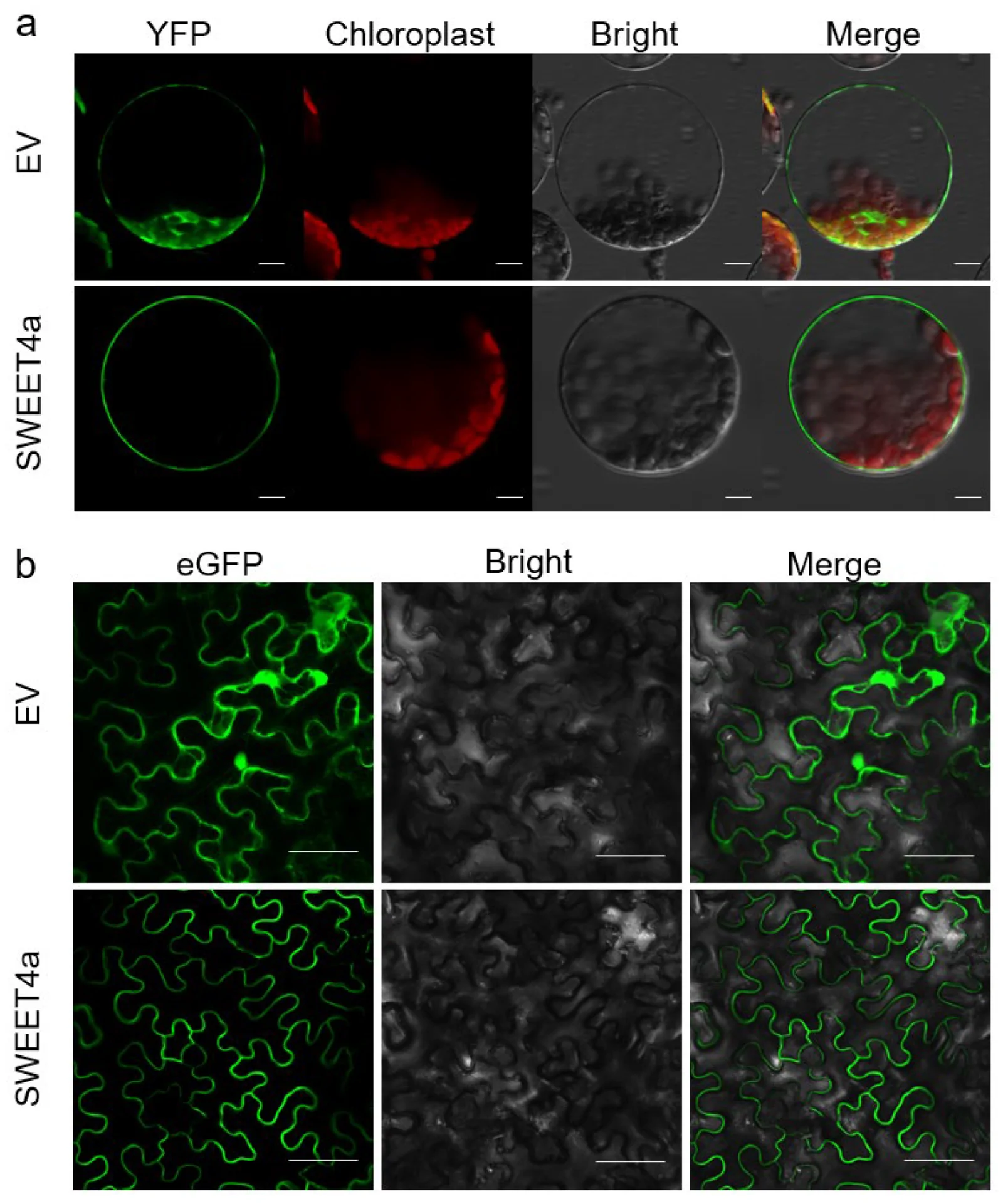
Subcellular localization of NnSWEET4a in *A. thaliana* protoplasts and *N. benthamiana* leaves. (**a**) Confocal images of *A. thaliana* protoplasts transiently expressing *pA7-YFP* (EV) or *NnSWEET4a-YFP* fusions (SWEET4a). (**b**) Confocal images of *N. benthamiana* epidermal leaf cells transiently expressing *NnSWEET4a: eGFP* fusions. Scale bar = 5 µm in (**a**); 50 µm in (**b**).

Although *NnSWEET11* exhibits a statistically significant upregulation under NaCl treatment, and NnSWEET15 confers salt sensitivity in yeast complementation assays, both genes display exceptionally low basal expression levels in lotus (Figure 4). Consequently, we focus on *NnSWEET4a* for further functional analysis. Firstly, we generated the C-terminal YFP fusion construct (*NnSWEET4a-YFP/pA7*) and C-terminal eGFP fusion construct (*NnSWEET4a-eGFP/p1300*), which were transiently expressed in both *A. thaliana* mesophyll protoplasts and *N. benthamiana* epidermal leaf cells, respectively. In both systems, reporter fluorescence exhibited a sharp, continuous signal outlining the cell periphery. This pattern starkly contrasted with the diffuse, nucleoplasmic and cytosolic distribution observed in control cells expressing free YFP or eGFP (Figure 6). These consistent results demonstrate that NnSWEET4a is targeted to the plasma membrane.

### 2.7. Overexpression of NnSWEET4a Compromises Salt Tolerance in A. thaliana

**Figure 7:**
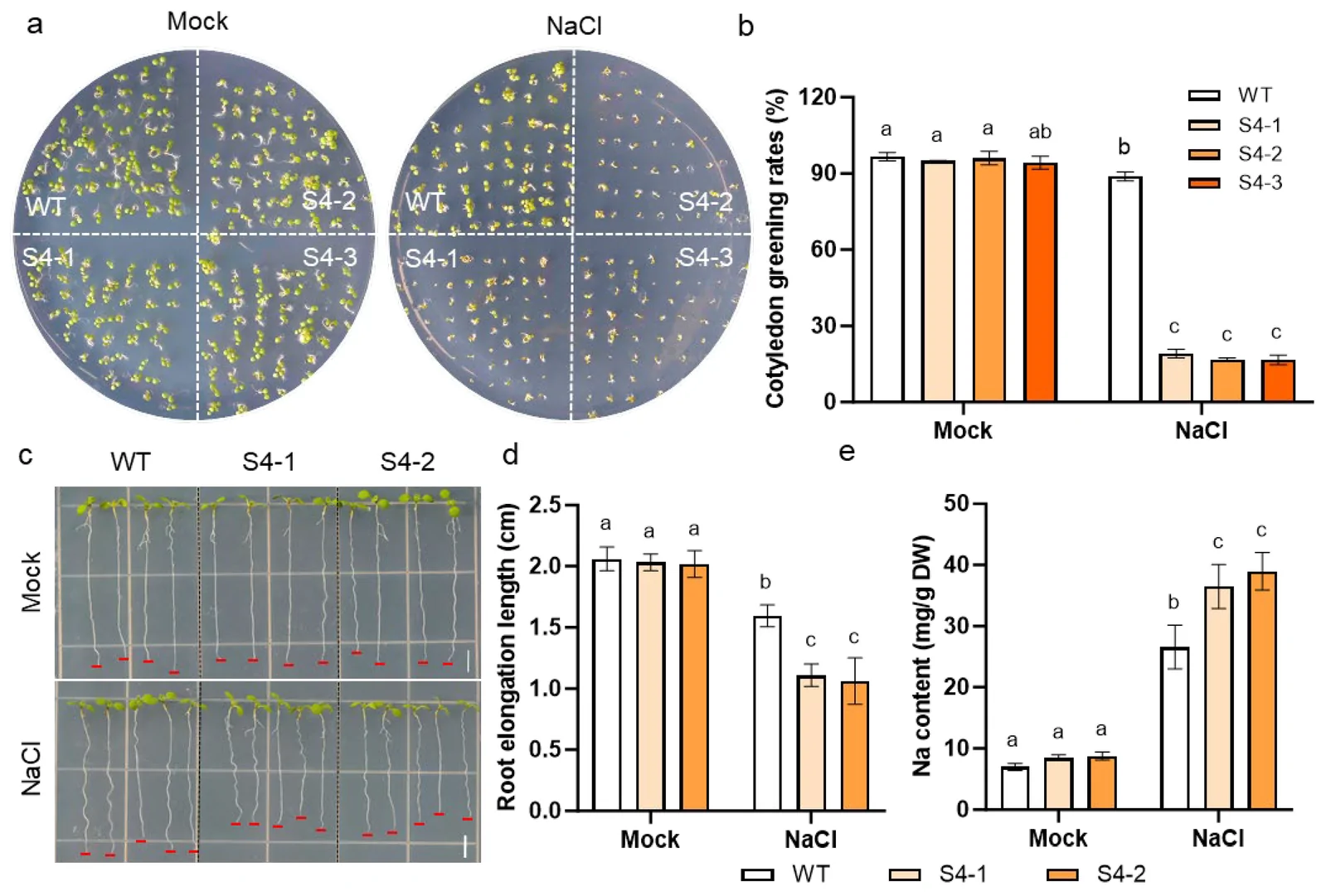
NnSWEET4a-induced salt sensitivity in *A. thaliana*. (**a**) Phenotypes of the wild type (WT), S4-1, S4-2 and S4-3 plants grown on ½ MS medium (containing 0.8% *w*/*v* sucrose) supplemented with 0 (Mock) or 150 mM NaCl (NaCl) for 4 d. (**b**) Quantitative analysis of cotyledon greening rates of plants in (**a**). (**c**–**e**) S4-1 and S4-2 plants are more sensitive than WT to NaCl. Four-day-old *A. thaliana* seedlings were transferred to 1/2 MS medium (containing 0.8% *w*/*v* sucrose) with 0 (Mock) and 80 mM NaCl (NaCl) and grown for another 3 d (**c**); root elongation was measured (**d**). Analysis of Na^+^ contents in WT and SWEET4a transformants (S4-1, S4-2) (**e**). Data are mean ± SD; *n* = 3 in (**b**), 8 in (**d**) and 4 in (**e**) for independent biological replicates; one-way ANOVA test: *p* < 0.05. Different lowercase letters indicate significant differences according to Tukey’s multiple comparison test.

Given that a genetic transformation system for lotus has not yet been established, we ectopically expressed *NnSWEET4a* in *A. thaliana* to investigate its role in salt stress responses in plants. Three independent homozygous transgenic lines exhibiting high-level expression were confirmed by qRT-PCR (Figure S6a). Under non-stress conditions, transgenic seedlings displayed wild-type (WT) morphology and growth. However, upon exposure to 150 mM NaCl, *NnSWEET4a*-overexpressing lines exhibited markedly reduced cotyledon greening rates (Figure 7a,b) and severely impaired primary root elongation (Figure 7c,d) relative to WT controls. Furthermore, under 80 mM NaCl treatment, transgenic plants accumulated significantly higher Na^+^ concentrations compared with WT plants (Figure 7e), despite having comparable basal Na^+^ levels under normal conditions. Collectively, these findings indicate that NnSWEET4a overexpression exacerbates salt sensitivity and promotes Na^+^ accumulation in plants.

### 2.8. Expression of SOS Pathway Was Altered in NnSWEET4a-Overexpressing Plant

**Figure 8:**
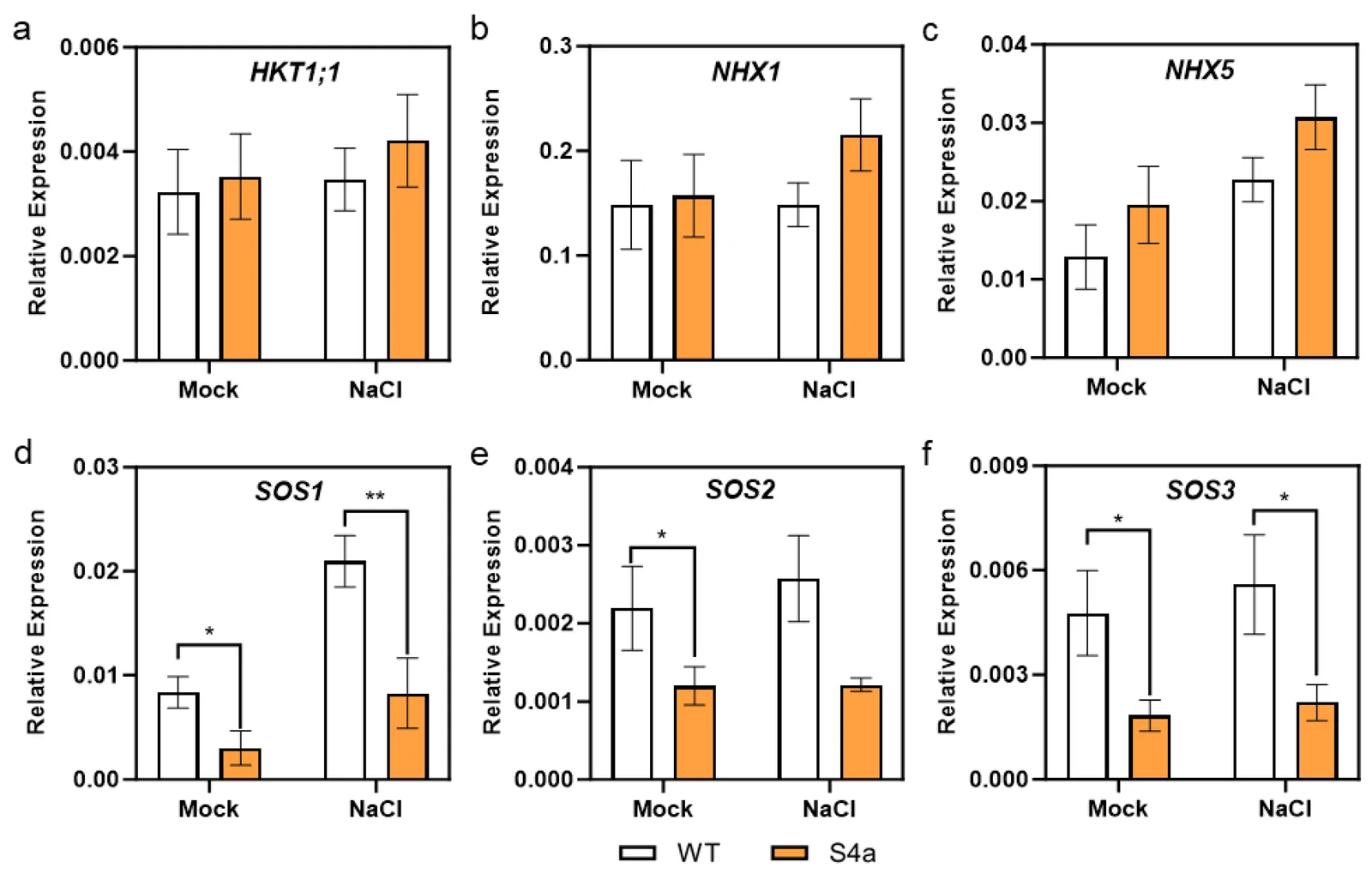
(**a**–**f**) Expressions of Na^+^ transport-related genes were determined by qRT-PCR in seedlings of WT and the *NnSWEET4a* transformant (S4a) cultured on ½ MS plates (containing 0.8% *w*/*v* sucrose) supplemented with 0 (Mock) and 150 mM NaCl (NaCl) for 4 d. *AtActin2* was used as an internal control. Data are mean ± SD; *n* = 3 for independent biological replicates; Student’s *t*-test: * *p* < 0.05 and ** *p* < 0.01.

To elucidate the molecular mechanism underlying the salt-hypersensitive phenotype in *Arabidopsis*, we quantified transcript levels of key regulators of Na^+^ homeostasis in transgenic and WT plants under control and salt-stressed conditions. Expression of endomembrane-localized Na^+^/H^+^ antiporters (AtNHX1 and AtNHX5) and the plasma membrane Na^+^ transporter AtHKT1;1 remained unchanged (Figure 8a–c), suggesting no major perturbation in vacuolar sequestration or root-to-shoot Na^+^ retrieval. In contrast, the three core components of the SOS pathway, including *SOS1* (plasma membrane Na^+^/H^+^ antiporter), *SOS2* (serine/threonine protein kinase) and *SOS3* (calcium sensor/CBL4), were significantly downregulated in *NnSWEET4a*-overexpressing lines under both control and salt-treated conditions (Figure 8d–f). This coordinated suppression implies that *NnSWEET4a* may interfere with the transcriptional level of the SOS pathway, thereby compromising Na^+^ extrusion capacity and salt tolerance. Interestingly, salt treatment also induced the expression of *NnSOS2* and *NnSOS3* in lotus (Figure S7).

### 2.9. Expression of NnSWEET15 Also Exhibited Enhanced Sensitivity to Salt Stress and Suppression of the SOSs in A. thaliana

**Figure 9:**
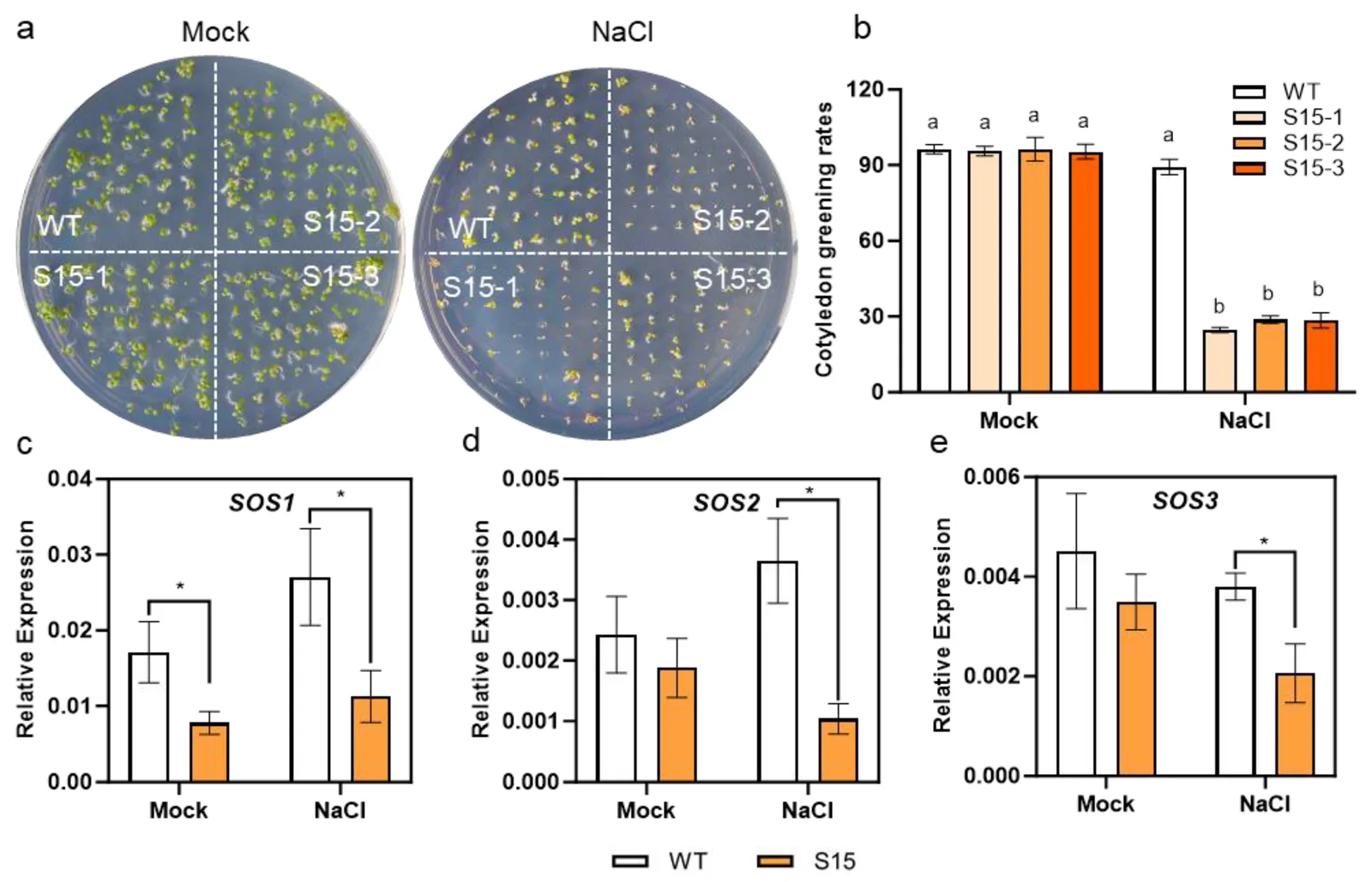
*NnSWEET15*-induced salt sensitivity in *A. thaliana*. (**a**) Phenotypes of the WT, S15-1, S15-2 and S15-3 plants grown on ½ MS medium (containing 0.8% *w*/*v* sucrose) supplemented with 0 (Mock) or 150 mM NaCl (NaCl) for 4 d. (**b**) Quantitative analysis of cotyledon greening rates of plants in (**a**). (**c**–**e**) Expressions of SOS pathway genes were determined by qRT-PCR in seedlings of WT and the *NnSWEET15* transformant (S15) cultured on ½ MS plates (containing 0.8% *w*/*v* sucrose) supplemented with 0 (Mock) and 150 mM NaCl (NaCl) for 4 d. *AtActin2* was used as an internal control. Data are mean ± SD, *n* = 3 in (**b**–**e**) for independent biological replicates; one-way ANOVA test for (**b**), *p* < 0.05, different lowercase letters indicate significant differences according to Tukey’s multiple comparison test; Student’s *t*-test for (**c**–**e**), * *p* < 0.05.

It remains unclear whether the downregulation of SOS pathway components is specific to *NnSWEET4a* or represents a general consequence of other *NnSWEET* family members being overexpressed. Given that NnSWEET15 exhibits substrate transport properties comparable to those of NnSWEET4a and confers analogous salt sensitivity in yeast, we generated *A. thaliana* transgenic lines overexpressing *NnSWEET15* for comparative phenotypic characterization (Figure S6b). Interestingly, *NnSWEET15*-overexpressing transgenic lines displayed phenotypes analogous to those of *NnSWEET4a*-overexpressing lines, including reduced cotyledon greening rates under salt stress (Figure 9a,b) and suppression of the transcriptional level of the SOS signaling pathway (Figure 9c–e). These results indicate that SOS pathway suppression is not exclusive to *NnSWEET4a* overexpression but likely represents a shared downstream consequence of disrupted sugar homeostasis.

### 2.10. Sugar Homeostasis Was Altered in NnSWEET4a-Overexpressing Plants Under Salt Stress

**Figure 10:**
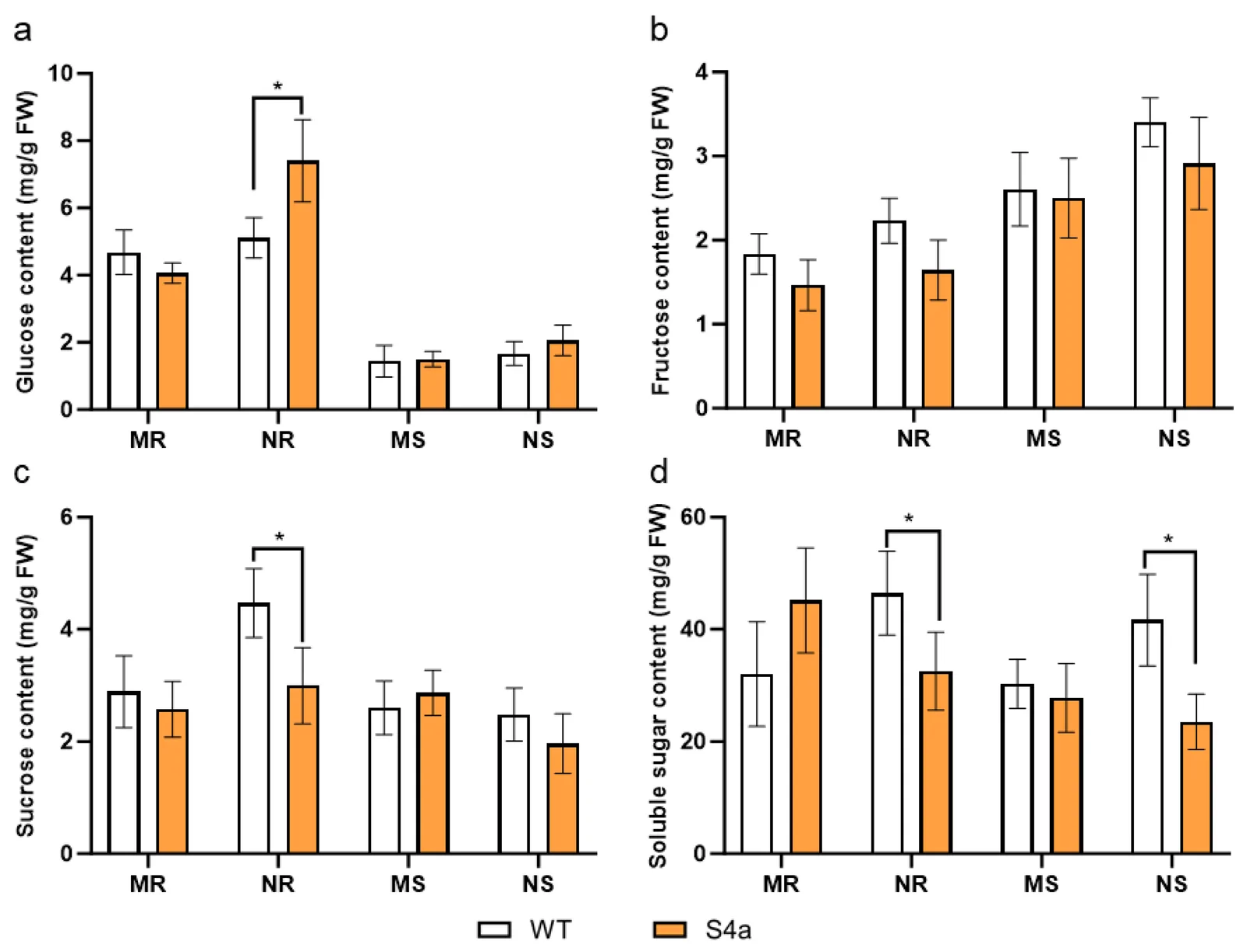
Sugar content in WT and *NnSWEET4a* transformant (S4a) seedlings. (**a**–**d**) Glucose (**a**), fructose (**b**), sucrose (**c**) and total soluble sugars (**d**) contents were measured in shoots and roots of 2-week-old seedlings grown in a hydroponic solution treated with or without 50 mM NaCl for 24 h. Data are mean ± SD; *n* = 4 in (**a**–**d**) for independent biological replicates; Student’s *t*-test: * *p* < 0.05. MR and MS: Roots and shoots from Mock; NR and NS: Roots and shoots from NaCl.

To verify the hypothesis, we detected the contents of glucose, fructose, sucrose and total soluble sugars in roots and shoots of 2-week-old WT and *NnSWEET4a*-overexpressing seedlings under control and 50 mM NaCl conditions. Under salt stress, *NnSWEET4a*-overexpressing roots accumulated significantly higher glucose concentrations relative to WT, whereas sucrose levels were markedly reduced (Figure 10a–c). Fructose content remained largely unchanged across both tissues and treatments. Notably, total soluble sugar levels were significantly reduced in both roots and shoots of *NnSWEET4a*-overexpressing seedlings compared with WT following NaCl treatment (Figure 10d).

### 2.11. Exogenous Sugars Exacerbate the Sensitivity of NnSWEET4a-Overexpressing Plants to Salt Stress

**Figure 11:**
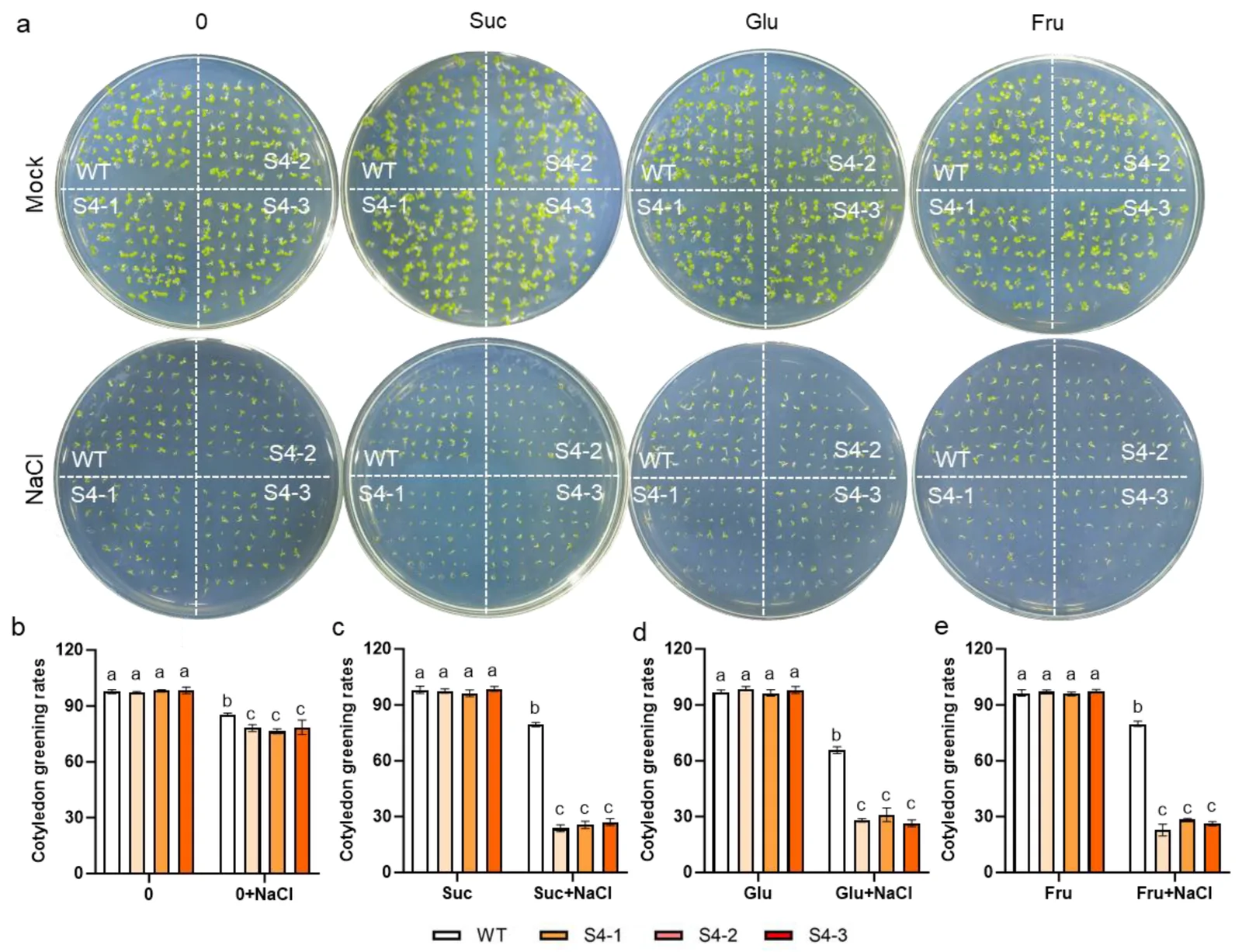
Sugar-dependent salt sensitivity induced by *NnSWEET4a* in *A. thaliana*. (**a**) Phenotypes of WT, S4-1, S4-2 and S4-3 seedlings cultured on ½ MS medium with different carbon sources (0, sugar-free or Suc; Glu; Fru for 0.8% *w*/*v*). The media were separately supplemented with 0 (Mock) or 150 mM NaCl (NaCl) for 4 d. (**b**–**e**) Statistical quantification of cotyledon greening rates. Data are mean ± SD; *n* = 3 for independent biological replicates; one-way ANOVA test: *p* < 0.05. Different lowercase letters indicate significant differences according to Tukey’s multiple comparison test.

To assess the functional dependence of the salt-hypersensitive phenotype on exogenous sugar availability, we evaluated cotyledon greening rates of WT and *NnSWEET4a*-overexpressing lines germinated on ½ MS medium supplemented with or without 150 mM NaCl and different carbon sources (sucrose, glucose, or fructose; 0.8% *w*/*v*) for a 4-day period. In the absence of salt, all genotypes exhibited comparable greening efficiency across all sugar-supplemented media (Figure 11). Under salt stress, a slight difference in greening was observed between WT and transgenic lines on sugar-free medium; however, exogenous sugars significantly exacerbate the sensitivity of *NnSWEET4a*-overexpressing plants to salt stress compared to WT (Figure 11).

### 2.12. Inhibition of the SOS Pathway by the Overexpression of NnSWEET4a Was Sugar-Dependent

**Figure 12:**
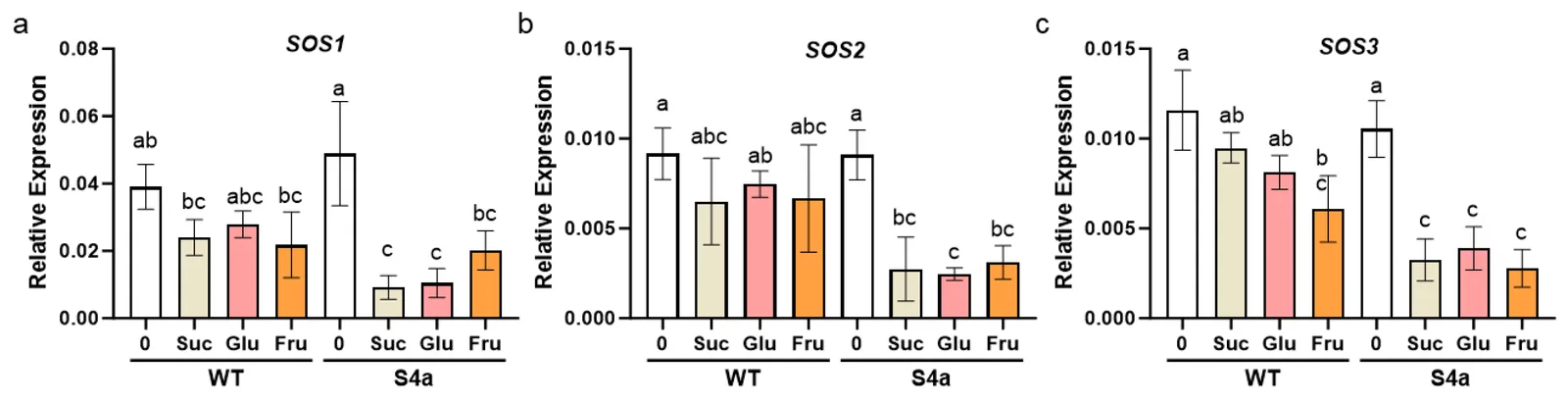
Expressions of *SOS* pathway genes were determined by qRT-PCR in seedlings of WT and S4a cultured on ½ MS plates with different carbon sources (0, sugar-free or Suc; Glu; Fru for 0.8% *w*/*v*) supplemented with 150 mM NaCl for 4 d. (**a–c**) Expression of *SOS1* (**a**); *SOS2* (**b**); *SOS3* (**c**). *AtActin2* was used as an internal control. Data are mean ± SD; *n* = 3 for independent biological replicates; one-way ANOVA test: *p* < 0.05. Different lowercase letters indicate significant differences according to Tukey’s multiple comparison test.

To further elucidate the mechanistic link between *NnSWEET4a* overexpression and SOS pathway modulation under salt stress, we quantified transcript abundances of *SOS1*, *SOS2*, and *SOS3* in WT and *NnSWEET4a*-overexpressing Arabidopsis seedlings grown on salt-containing media with or without exogenous sugars. In the absence of added sugars, *NnSWEET4a* overexpression did not significantly alter *SOS* gene expression relative to WT under salt stress. Similarly, exogenous sugar supplementation exerted no statistically significant effect on *SOS* transcript abundance in WT plants under identical salt conditions. In contrast, the addition of sugar greatly reduced the expression of *SOS1*, *SOS2*, and *SOS3* in *NnSWEET4a*-overexpressing plants under salt stress compared to the corresponding sugar-free salt control (Figure 12).

## 3. Discussion

SWEET proteins are a class of conserved sugar transporters widely present in plants, playing pivotal roles in growth and development, plant–pathogen interactions and abiotic stress response [41,42]. In this study, a total of 14 *NnSWEET* genes were identified in lotus, which is fewer than the number in *A. thaliana* (17) and *O. sativa* (21) [13,14]. This interspecies divergence likely reflects genetic diversity and differential evolutionary selection pressures among plants (Table 1, Figure 1). The NnSWEET proteins exhibit significant variations in amino acid length, molecular weight, isoelectric point, and stability, which may be related to their functional diversification (Table 1). Phylogenetic analysis showed that NnSWEET proteins are divided into four clades (I–IV), which is consistent with the previous classification of SWEET proteins from other plant species [13,14] (Figure 1). Most *NnSWEET* genes are distributed on chromosomes 1 and 3, with chromosome 3 harboring the largest number (7) (Figure S1), which is similar to the uneven distribution pattern of *SWEET* genes in *A. thaliana* and rice. Tissue-specific expression analysis revealed that all *NnSWEET* genes are expressed in at least one lotus tissue, suggesting their universal involvement in lotus growth and development (Figure S3). Meanwhile, we identified five pairs of segmental duplication events in lotus and four pairs in *Nelumbo lutea* (Figure S4), indicating that segmental duplication may be the main mechanism for the expansion of the NnSWEET family in *Nelumbo* species [43]. Furthermore, the Ka/Ks ratios of all duplicated gene pairs are less than one (Table S2), indicating that the *NnSWEET* genes underwent purifying selection during evolution, which is conducive to maintaining their conservative functions in sugar transport [44].

The sugar transport activity assay using the hexose transporter-deficient yeast strain *EBY.VW4000* revealed distinct substrate specificities among NnSWEET members. NnSWEET1b, NnSWEET2, NnSWEET4a, NnSWEET4b and NnSWEET15 exhibit broad-spectrum transport activity for glucose, mannose, fructose, or sucrose, which may be related to their role in mediating sugar transport in various tissues and under different physiological conditions. In contrast, NnSWEET3 and NnSWEET10 failed to support yeast growth on any tested sugars, which may be due to their localization in the endomembrane system (e.g., endoplasmic reticulum); the yeast heterologous expression system is not suitable for detecting the transport activity of non-plasma membrane-localized SWEET proteins. Their actual transport properties need to be verified using plant-based systems or in vitro transport assays [16,45]. Other NnSWEET members like NnSWEET1a, NnSWEET11, and NnSWEET16 display distinct substrate preferences, which may reflect their functional specialization in specific sugar transport processes, further supporting the functional diversification of the NnSWEET family (Figure 2).

Salt stress is a major abiotic stress limiting lotus growth and productivity [46], and *SWEET* genes have been reported to be involved in salt tolerance regulation in various plants [12,22,23,24,25,26,27,40]. Promoter cis-acting element analysis showed that stress-responsive elements are significantly enriched, indicating that *NnSWEET* genes may play important roles in lotus stress adaptation (Figure 3). Inducible expression analysis showed that among the detected genes, *NnSWEET4a* was significantly upregulated (~three-fold) under NaCl treatment, implying that it may be a key responsive gene of lotus to salt stress (Figure 4). Additional yeast functional assays revealed that yeast strains heterologously expressing *NnSWEET4a* or *NnSWEET15* displayed heightened sensitivity under high-salt conditions (Figure 5). This phenotypic pattern resembles that of the well-characterized AtSWEET15, a salt-inducible gene reported to negatively modulate plant salt tolerance. [12]. NnSWEET4a was localized on the plasma membrane, which is a prerequisite for its involvement in sugar transport and salt stress response, as plasma membrane-localized transporters can directly mediate substance exchange between cells and the external environment (Figure 6). Overexpression of *NnSWEET4a* did not affect plant growth under normal conditions but significantly reduced salt tolerance under NaCl treatment, as evidenced by the decreased cotyledon greening rate, impaired primary root elongation, and increased Na^+^ accumulation in seedlings (Figure 7).

**Figure 13:**
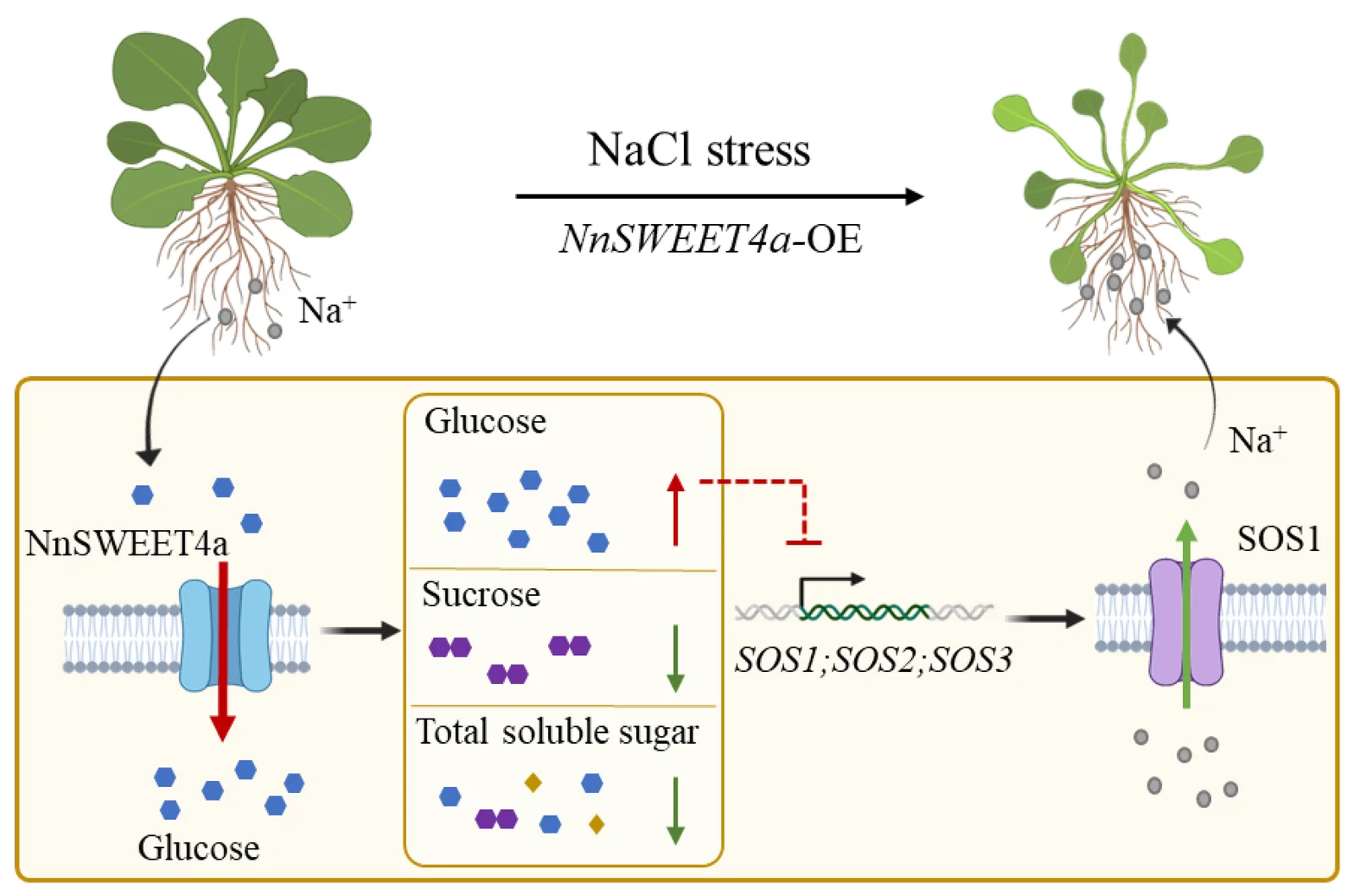
Proposed working model for *NnSWEET4a*-mediated alterations in sugar homeostasis and salt hypersensitivity under salinity. Under salt stress, overexpression of *NnSWEET4a* leads to glucose accumulation accompanied by reduced sucrose and total soluble sugar levels. Elevated glucose could repress the transcript levels of *SOS* genes (*SOS1*, *SOS2*, and *SOS3*), thereby impairing Na^+^ extrusion and resulting in plant salt hypersensitivity. Red arrows indicate increased levels, whereas green arrows indicate decreased levels.

Plants employ two primary adaptive strategies to mitigate osmotic stress induced by salinity: (i) modulation of ion transport, including ion uptake, extrusion, and intracellular sequestration, and (ii) metabolic regulation of osmolytes [47]. The expressions of *AtNHX1*, *AtNHX5* (endomembrane Na^+^/H^+^ antiporters) and *AtHKT1;1* (plasma membrane Na^+^ transporter) remained unchanged in *NnSWEET4a*-overexpressing plants [48,49,50] (Figure 8a–c), suggesting that expression of *NnSWEET4a* does not affect vacuolar Na^+^ sequestration or root-to-shoot Na^+^ retrieval. In contrast, the transcript levels of the three core components of the *SOS* pathway (*SOS3-SOS2-SOS1*) were significantly downregulated in *NnSWEET4a*-overexpressing lines under both normal and salt stress conditions (Figure 8d–f). The SOS pathway is a key regulatory pathway for plant salt tolerance, in which SOS1 mediates Na^+^ extrusion from cells, and SOS2 and SOS3 regulate SOS1 activity [51,52,53]. Notably, transgenic plants overexpressing *NnSWEET15* recapitulated the same phenotypic and molecular responses, including heightened salt sensitivity and suppression of *SOS* pathway genes (Figure 9), indicating that these effects reflect a shared physiological consequence of disrupted sugar homeostasis rather than *NnSWEET4a*-specific interactions. Consistently, sugar homeostasis was extensively altered due to the expression of *NnSWEET4a*, and both the salt sensitivity and SOS pathway inhibition were enhanced in sugar-supplemented growth media (Figure 10, Figure 11 and Figure 12), confirming their dependence on altered sugar status. These observations support the speculation that NnSWEET4a-induced salt hypersensitivity may arise indirectly as a downstream consequence of sugar homeostasis disruption, rather than through direct physical or regulatory interaction with SOS pathway components (Figure 13).

Under salt stress, seedlings overexpressing *NnSWEET4a* exhibited significantly elevated root glucose concentrations, concomitant with reduced sucrose and total soluble sugar levels relative to wild-type (WT) controls (Figure 10). Exogenous glucose application consistently enhanced salt sensitivity in *NnSWEET4a*-overexpressing lines (Figure 11), supporting a functional link between glucose accumulation and compromised salt tolerance. The glucose-induced salt sensitivity phenotype was also observed in other species [54,55]. Exogenous supplementation with sucrose or fructose also exacerbated salt sensitivity and SOS pathway inhibition in these transgenic plants (Figure 11 and Figure 12). It is speculated that *NnSWEET4a* overexpression may promote the metabolic conversion of these sugars into glucose. This inference is further corroborated by the observed decline in root sucrose and total soluble sugar pools under salinity (Figure 10). SWEET proteins have also been reported to potentially mediate the conversion of other sugars into glucose under diverse stresses [56,57]. While the precise enzymatic or regulatory mechanisms underlying NnSWEET4a-mediated sugar interconversion, and the downstream signaling by which elevated glucose suppresses SOS pathway gene expression, remain to be elucidated, our findings establish a mechanistic framework wherein NnSWEET4a impairs salt tolerance via disruption of sugar homeostasis. This model provides a foundational basis for future investigations into SWEET-dependent regulation of sugar homeostasis and salt stress adaptation.

## 4. Materials and Methods

### 4.1. Plant Materials, Transformation and Phenotyping

Lotus plants (cultivar Cunsanlian) were collected from Xiangtan “Cunsanlian” Breeding and Protection Base. The lotus seedlings were hydroponically cultivated in Hoagland solution under controlled growth chamber conditions (16 h light/8 h dark photoperiod at 30 °C) for NaCl exposure experiments. *A. thaliana* (ecotype Columbia-0) was employed for functional characterization of *NnSWEET4a* and *NnSWEET15* via floral-dip-mediated Agrobacterium transformation. The full-length coding sequences of *NnSWEET4a* and *NnSWEET15* were cloned into the *pCAMBIA1300* vector containing the CaMV 35S promoter and eGFP reporter gene (primers are listed in Table S1). For salt tolerance phenotyping assays, surface-sterilized Arabidopsis seeds were subjected to vernalization and subsequently sown on horizontal half-strength Murashige and Skoog (½ MS) agar plates. These plates were supplemented with distinct carbon sources (sugar-free or sucrose, glucose, and fructose 0.8% *w*/*v*) plus 150 mM NaCl, and seedlings were cultured for 4 days [58]. Alternatively, seeds were sown on vertical ½ MS plates without NaCl; after germination, seedlings were transferred to new agar plates containing 80 mM NaCl and cultured for another 3 days [59]. The cotyledon greening rate and primary root elongation length were quantified as the primary phenotypic metrics of NaCl tolerance.

### 4.2. Identification and Physical Properties Analysis of NnSWEET Genes in N. nucifera

The lotus genome data used in this study are available under the Nelumbo Genome Database (http://nelumbo.cngb.org/nelumbo, accessed on 10 April 2026) [38]. All protein sequences from the lotus genome were scanned by HMMER 3.0 using a hidden Markov model (HMM) of the MtN3/saliva domain (PF03083), downloaded from Pfam (http://pfam.xfam.org/, accessed on 12 April 2026), with an e-value < 10^−5^. Protein molecular weight (Mw) and theoretical isoelectric point (pI), instability index, Aliphatic index, and grand average of hydropathicity (GRAVY) were calculated by Expasy (http://web.expasy.org/protparam/, accessed on 14 April 2026). The Cell-PLoc was used for the identification of the subcellular localization of candidate NnSWEETs [60].

### 4.3. Gene Structure, Conserved Motif, and Phylogenetic Analysis of NnSWEET Genes

The exon/intron structures of *NnSWEET* genes were analyzed using GSDS based on the genomic and coding sequence (http://gsds.cbi.pku.edu.cn/, accessed on 15 April 2026). The conserved motifs of full-length NnSWEET proteins were identified using MEME (https://meme-suite.org/, accessed on 15 April 2026). Multiple sequence alignment was carried out considering the full-length SWEET protein sequences from *A. thaliana* (https://www.arabidopsis.org/, accessed on 15 April 2026), *O. sativa* (https://www.phytozome.net/, accessed on 15 April 2026) and *N. nucifera* using the ClustalW program. An unrooted neighbor-joining (NJ) tree was constructed by MEGA 7.0 with 1000 bootstrap replicates and was displayed with iTOL (https://itol.embl.de/, accessed on 15 April 2026) [61].

### 4.4. Analysis of Chromosomal Distribution, Gene Duplication and Cis-Regulatory Element

The information regarding the locations of *NnSWEET* genes was obtained from the GFF genome annotation of *N. nucifera*, and the chromosomal visualization of *NnSWEET* genes was generated using Tbtools (version 2.471) [62]. The duplication events and synteny of the *NnSWEETs* between lotus and *N. lutea*, lotus and *A. thaliana*, and lotus and *O. sativa* were analyzed using MCScanX (http://chibba.pgml.uga.edu/mcscan2/, accessed on 15 April 2026). The gene duplication and collinearity were shown using Circos (version 0.69-8) and Tbtools software with default parameters [63]. In addition, the ratio of non-synonymous to synonymous nucleotide substitutions (Ka/Ks) was evaluated among duplicated gene pairs to detect the selection mode by Ka/Ks Calculator and ParaAT2.0 [64]. The sequences of 2000 bp from the promoter region of each *NnSWEET* gene were extracted and submitted to the PlantCARE database (https://bioinformatics.psb.ugent.be/webtools/plantcare/html/, accessed on 15 April 2026) to predict the cis-regulatory elements. The number of corresponding cis-regulatory elements was visualized using ChiPlot (https://www.chiplot.online/, accessed on 15 April 2026).

### 4.5. Expression Analysis of Genes and Verification by Quantitative Real-Time PCR (qRT-PCR)

Based on the published transcriptome data of lotus downloaded from the Nelumbo Genome Database (http://nelumbo.cngb.org/nelumbo/tools/expressionVisualization, accessed on 14 April 2026), the expression of *NnSWEET* genes in various tissues from all developmental stages was explored. Normalized gene expression values expressed as Fragments Per Kilobase of transcript per Million (FPKM) were transformed using log_2_ FPKM, and the heatmap was drawn with TBtools [65]. Total RNA was extracted using TRIzol reagent (Invitrogen, Carlsbad, CA, USA). cDNAs were synthesized using oligo(dT) primers and PrimeScript RT Enzyme Mix I (TaKaRa). qRT-PCR was performed on a Real-Time PCR Detection System (Bio-Rad, Hercules, CA, USA) using TBGreen Premix Ex Taq (Takara, Kusatsu, Japan) [66,67]. Expressions of putative *NnSOS1* (Nn4g24593), *NnSOS2* (Nn8g39476), and *NnSOS3* (Nn1g00242) were obtained by BLAST (version 2.14.0, NCBI, Bethesda, MD, USA) against Arabidopsis *SOS* sequences in lotus roots after 24 h of 50 mM NaCl treatment. *NnActin*, *ScActin* and *AtActin2* were used as the internal control for lotus, yeast and *A. thaliana*, respectively. Data analysis was performed employing the 2^−ΔΔCT^ method to calculate Ct values with three biological replicates. The primers used in this study are listed in Table S1.

### 4.6. Substrate Specificity Analysis of NnSWEETs in Yeast

The coding sequences of selected *NnSWEET* genes were amplified and ligated into the yeast expression vector pDR196 (primers for the yeast construct are listed in Table S1). For the yeast transport assays, the *pDR196-NnSWEET* constructs and the empty vector (EV) were transformed separately into the *Saccharomyces cerevisiae* strain *EBY.VW4000*, which was deficient in hexose transport [39]. Successfully transformed yeast cells were selected on a synthetic deficient medium without uracil (SD/-Ura) with 2% (*w*/*v*) maltose as the sole carbon source. Positive transformants of *pDR196-NnSWEETs* and EV were serially diluted (1×, 10×, 100×, and 1000×) and spotted onto SD/-Ura solid medium with 2% (*w*/*v*) maltose, 2% (*w*/*v*) glucose, 2% (*w*/*v*) mannose, 2% (*w*/*v*) fructose, and 2% (*w*/*v*) sucrose, respectively. Growth of all transformants was photographed after 3 d of incubation at 30 °C [68]. For yeast salt tolerance assays, yeast strains transformed with *NnSWEET* genes or EV were subjected to serial dilution gradients consistent with the method mentioned above. Subsequently, equal volumes of diluted yeast suspensions were spotted onto SD agar plates supplemented with or without 300 mM NaCl [69,70].

### 4.7. Subcellular Location Analysis of NnSWEET4a

The coding sequence of the *NnSWEET4a* gene (without stop codons) was amplified and cloned into the *pA7-YFP* and *pCAMBIA1300-eGFP* vectors (primers are listed in Table S1). *A. thaliana* mesophyll protoplasts were freshly isolated from fully expanded rosette leaves of 4-week-old wild-type seedlings following a standard enzymatic digestion method. The purified protoplasts were then transfected with the *NnSWEET4a*-YFP/pA7 plasmid via polyethylene glycol (PEG)-mediated transformation [71]. For transient expression in *N. benthamiana* leaves, the recombinant plasmid and the empty vector were introduced to *Agrobacterium tumefaciens* GV3101 and transfected into tobacco leaves with a syringe [72]. After two days post-infiltration, samples were taken from the tobacco leaves, and the subcellular localization of NnSWEET4a was visualized using confocal laser scanning microscopy (CSIM130, Sunny, Beijing, China).

### 4.8. Measurement of Na^+^ Content

Arabidopsis transformant seedlings were harvested, rinsed sequentially with ultrapure water, a 25 mM CaCl_2_ solution (pH 5.0) and ultrapure water again to remove apoplastic Na^+^ ions, followed by drying at 80 °C. Dried samples were digested in 70% (*v*/*v*) ultrapure nitric acid at 100 °C for 2 h using a water bath, cooled to room temperature, and diluted with ultrapure water [73,74]. Total Na^+^ concentration was determined by inductively coupled plasma mass spectrometry (ICP-MS, NexION 300, PerkinElmer, Waltham, MA, USA).

### 4.9. Determination of Glucose, Fructose, Sucrose and Total Soluble Sugar Content

Two-week-old wild-type (WT) and *NnSWEET4a* transgenic Arabidopsis seedlings were cultured hydroponically. Following a 24 h treatment with either 0 or 50 mM NaCl, shoots and roots were harvested separately to quantify sugar concentrations. All the samples were quantified using a glucose–fructose–sucrose kit (G0545F, Grace Biotechnology, Suzhou, China). The principle of the kit is as follows: sucrose and fructose are converted to glucose by the action of specific enzymes, and glucose simultaneously reduces NADP^+^ to NADPH by the action of enzyme complexes such as hexokinase. The contents of sucrose, glucose, and fructose are calculated by measuring the increase in NADPH at 340 nm. The soluble sugar content was detected by the plant soluble sugar content assay kit according to the manufacturer’s protocol (BC0030, Solarbio, Beijing, China). All experiments were conducted using four biological replicates.

### 4.10. Statistical Analysis

Details of statistical analyses are presented in the figure legends. All experimental data were taken from at least three independent experiments. Statistical analyses were performed on GraphPad Prism software (version 8.4.0, USA). The significance of the study was calculated using a two-tailed Student’s *t*-test for two groups and a one-way ANOVA test followed by Duncan’s multiple range test. Statistical significance was defined at *p* < 0.05 throughout this study.

## 5. Conclusions

This study provides a comprehensive genome-wide analysis of the *SWEET* gene family in *N. nucifera*, identifying 14 NnSWEET members and characterizing their chromosomal localization, gene structures, conserved motifs, phylogenetic and collinear relationships, promoter cis-acting elements and sugar transport activities. The tissue-specific expression patterns and salt-induced characteristics of *NnSWEET* genes indicate the functional diversity and potential mechanisms underlying salt-stress responses. Arabidopsis plants with ectopic overexpression of *NnSWEET4a* exhibited salt-sensitive phenotypes, and its regulatory function may be exerted through the SOS signaling pathway in a sugar-dependent manner (Figure 13). Our findings provide a foundation for further investigation into the functional properties of the *NnSWEET* gene family, particularly its role in salt-stress adaptation.

## Figures and Tables

**Table 1:** Basic characteristics of sequence length, molecular weight, isoelectric point and predicted subcellar localizations for SWEET proteins in lotus.

Gene-ID	Length (aa)	Molecular Weight (kDa)	Theoretical pI	Instability Index	Aliphatic Index	GRAVY	Predicted Subcellular Localization
*NnSWEET1a*	242	26.56	9.11	37.72	110.79	0.705	Cell membrane.
*NnSWEET1b*	254	28.45	9.54	36.29	109.33	0.623	Cell membrane; Endoplasmic reticulum.
*NnSWEET1c*	255	28.11	9.82	44.22	105.18	0.499	Cell membrane.
*NnSWEET2*	235	25.92	8.93	46.79	119.87	0.898	Cell membrane; Endoplasmic reticulum.
*NnSWEET3*	250	27.93	9.27	40.22	116.56	0.530	Endoplasmic reticulum.
*NnSWEET4a*	264	29.22	8.73	38.76	123.33	0.686	Cell membrane.
*NnSWEET4b*	265	29.38	9.08	38.99	124.26	0.712	Cell membrane.
*NnSWEET8*	239	26.59	9.02	36.27	126.36	0.855	Cell membrane.
*NnSWEET10*	292	32.59	8.27	37.20	114.79	0.535	Endoplasmic reticulum.
*NnSWEET11*	287	32.83	9.07	38.47	117.14	0.545	Endoplasmic reticulum.
*NnSWEET12*	276	30.89	9.39	34.83	120.43	0.681	Cell membrane.
*NnSWEET15*	270	30.42	8.98	33.35	115.93	0.579	Cell membrane.
*NnSWEET16*	358	39.22	9.79	40.26	110.22	0.290	Tonoplast; Endoplasmic reticulum.
*NnSWEET17*	248	27.12	6.30	35.19	115.60	0.656	Tonoplast; Cell membrane.

## Data Availability

The original contributions presented in this study are included in the article/Supplementary Material. Further inquiries can be directed to the corresponding author.

## Supplementary Materials

The following supporting information can be downloaded at https://www.mdpi.com/article/10.3390/plants15162542/s1. Figure S1: Chromosomal locations of *SWEET* genes.; Figure S2: Conserved motif and gene structure analysis of the *NnSWEETs* in lotus; Figure S3: Expression patterns of *NnSWEET* genes in different tissues; Figure S4: Evolutionary relationship of lotus *SWEET* genes; Figure S5: Ectopic expression of *NnSWEET3* and *NnSWEET10* in yeast; Figure S6: Ectopic expression of *NnSWEET4a* and *NnSWEET15* in Arabidopsis; Figure S7: Expression of putative *NnSOS1*, *NnSOS2* and *NnSOS3* in lotus roots under 50 mM NaCl treatment for 24 h detected by qRT-PCR; Table S1: Primer sequences used for qRT-PCR validation and vector construction; Table S2: The ka/ks of the syntenic gene pairs SWEETs in different species.
