# Supplementary material for: Overexpression of Lotus NnSWEET4a Alters Sugar Homeostasis and Induces Salt Hypersensitivity in Arabidopsis
Source: Plants (Basel). 2026 Aug 21;15(16):2542. doi: 10.3390/plants15162542 (PMC13516211; doi:10.3390/plants15162542)
Supplement: Supplementary file 1 [file plants-15-02542-s001.zip › SI-NnSWEET-figure-F.pdf]

Overexpression of Lotus *NnSWEET4a* Alters Sugar Homeostasis and Induces Salt Hypersensitivity in *Arabidopsis*

Shilong Zhao [1,†], Xiangxin Lu [1,†] , Zongyue Li [1], Xiaoyi Zhang [1], Siying Chen [1], Yan Gao [1], Jiashi Peng [1,2] and Tianyu Gu [1,*]

[1] Hunan Key Laboratory of Economic Crops Genetic Improvement and Integrated Utilization, Hunan University of Science and Technology, Xiangtan 411201, China
[2] Hunan Provincial Engineering Research Center for the Deep Processing and Health Benefits of Xianglian, School of Life and Health Sciences, Hunan University of Science and Technology, Xiangtan 411201, China
* Correspondence: guty@hnust.edu.cn
† These authors contributed equally to this work.

Supplementary material:

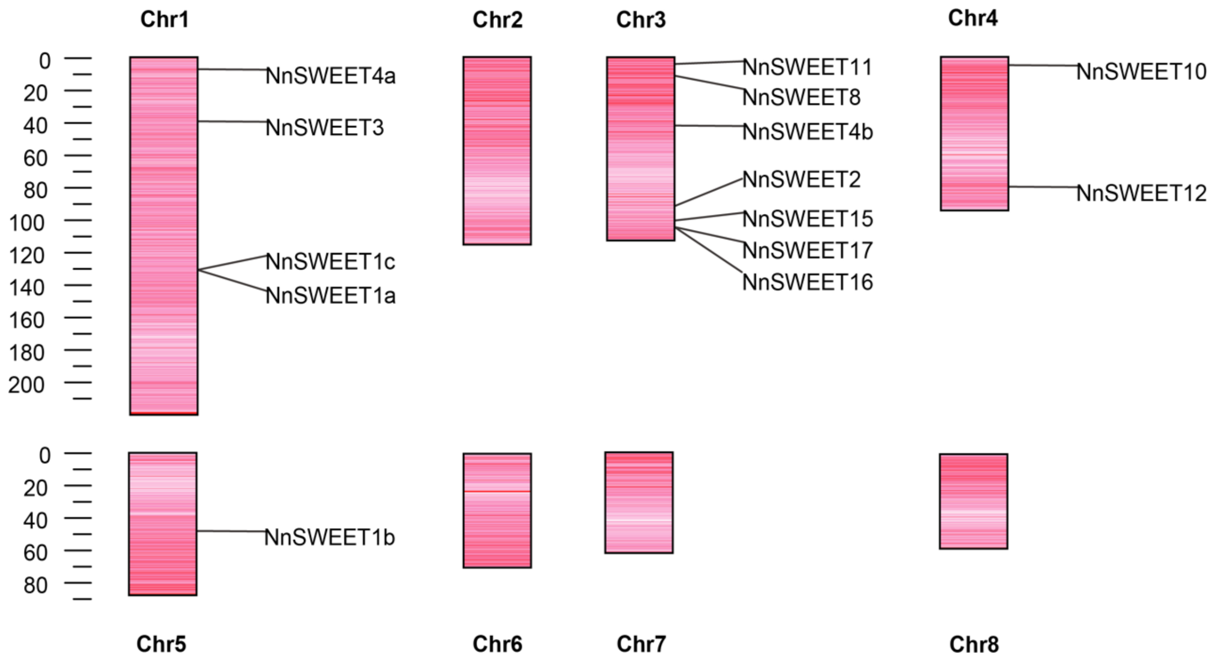

Figure S1. Chromosomal locations of *SWEET* genes. The scale on the left was used to estimate the length of the chromosomes (Mb). The *SWEET* genes of lotus were distributed on four chromosomes. Chr, chromosome.

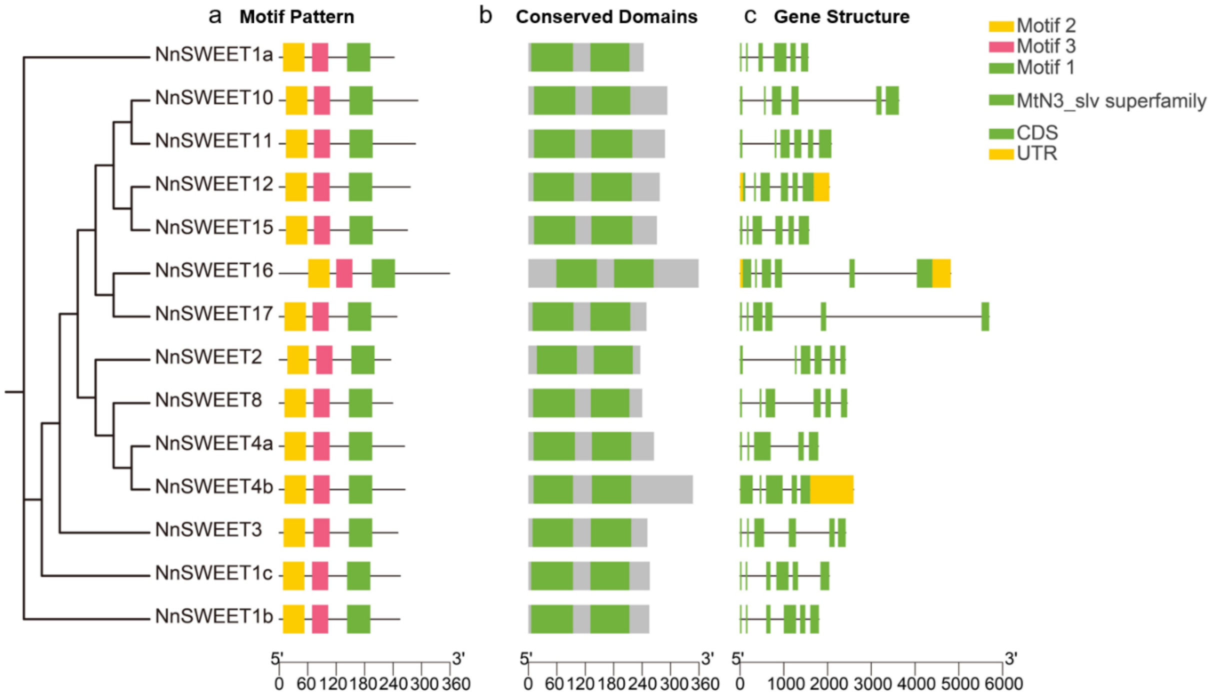

Figure S2. Conserved motif and gene structure analysis of the *NnSWEETs* in lotus. (a) Conserved motifs identified by MEME. Each colored box represents a motif. (b) Conserved domain structures of NnSWEET proteins. (c) Exon-intron structure of *NnSWEET* genes. The green and yellow boxes indicate CDS and UTR, respectively. The three analyses were integrated using Gene Structure View in TBtools software (v2.471).

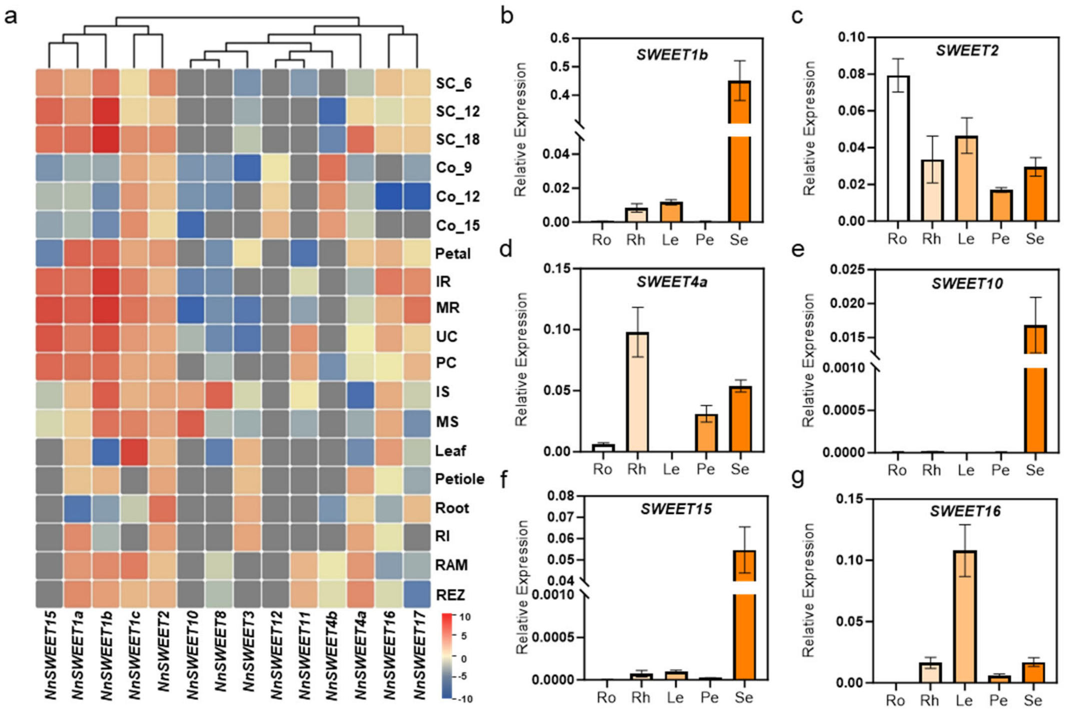

Figure S3. Expression patterns of *NnSWEET* genes in different tissues. (a) Heatmap for gene expression of 14 *NnSWEETs* in various tissues of N. nucifera. SC_6, Seed coat in 6 days; SC_12, Seed coat in 12 days; SC_18, Seed coat in 18 days; Co_9, Cotyledon in 9 days; Co_12, Cotyledon in 12 days; Co_15, Cotyledon in 15 days; IR, Immature Receptacle; MR, Mature Receptacle; UC, Unpollinated Carpel; PC, Pollinated Carpel; IS, Immature Stamen; MS, Mature Stamen; RI, Rhizome Internode; RAM, Rhizome Apical Meristem REZ, Rhizome Elongation Zone. The color scale represents log2 of the FPKM values. (b) Validation of the expression patterns of partial *NnSWEET* genes in different lotus tissues by qRT-PCR. Ro, Root; Rh, Rhizome; Le, Leaf; Pe, Petiole; Se, Seed. *NnActin* was used as an internal control. Data are mean ± SD, n = 3 for independent biological replicates.

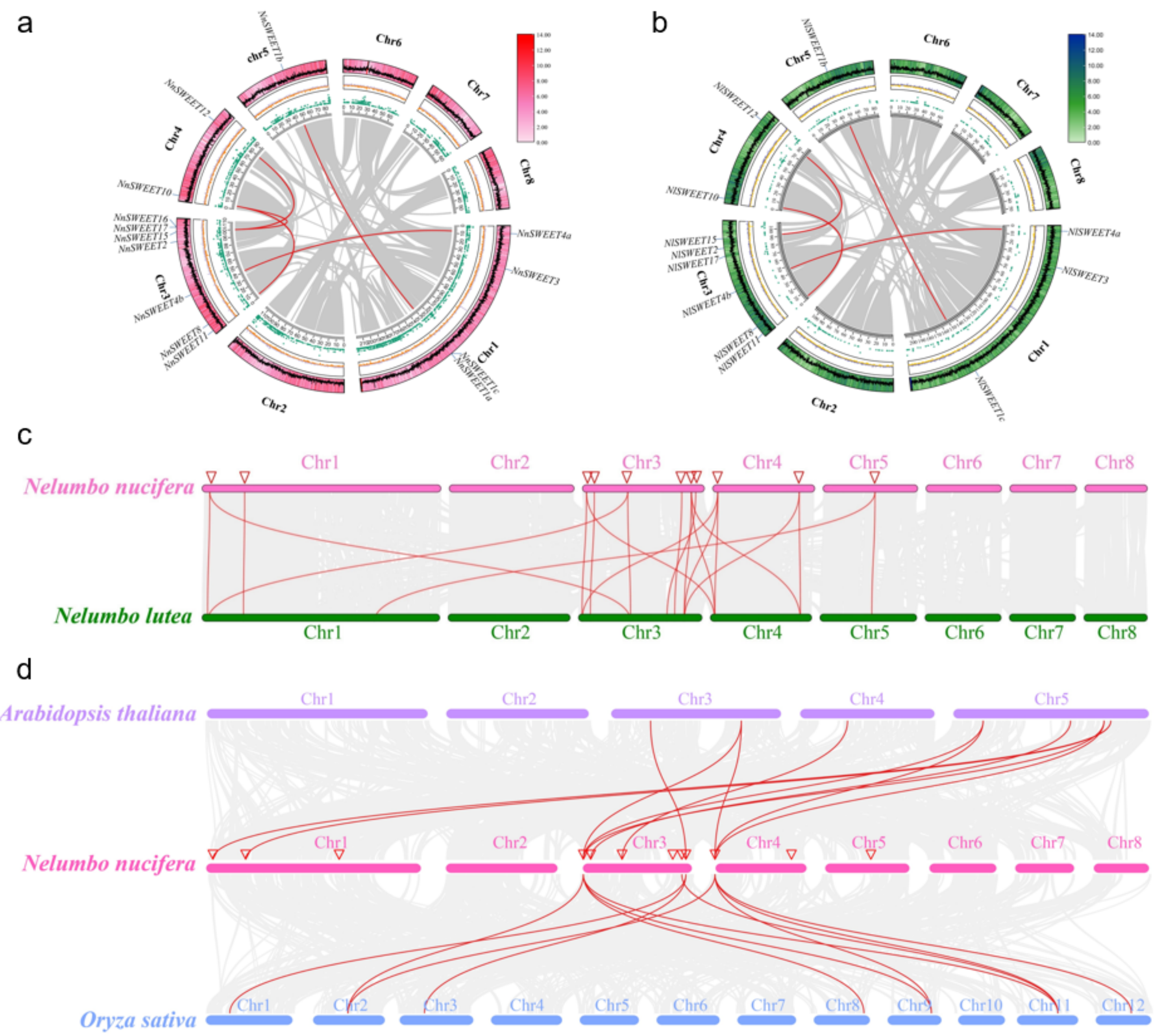

Figure S4. Evolutionary relationship of lotus *SWEET* genes. (a, b) Synteny analysis of *NnSWEET* (a) and *NlSWEET* (b) genes. The grey background lines and the red lines highlights genome collinearity modules and homologous gene pairs, respectively. (c) Synteny analysis of *SWEET* genes between *N. nucifera* and *N. lutea* genomes. (d) Synteny analysis of *SWEET* genes between *N. nucifera* and *A. thaliana* or *O. sativa.* The red lines indicated syntenic *SWEET* gene pairs.

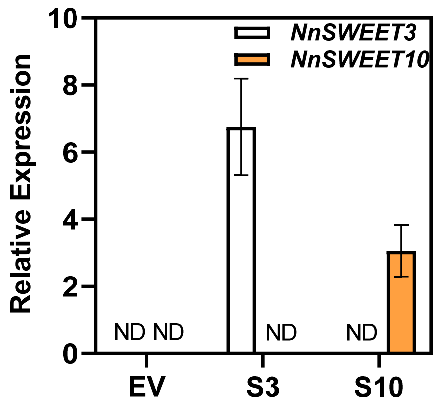

Figure S5. Ectopic expression of *NnSWEET3* and *NnSWEET10* in yeast. Yeasts harboring PDR196-NnSWEET3 (S3) or PDR196-NnSWEET10 (S10) were sampled to determine the relative expression of *NnSWEET3* and *NnSWEET10*. *ScActin* was used as an internal control. Data are means ± SD, n = 3 for independent biological replicates.

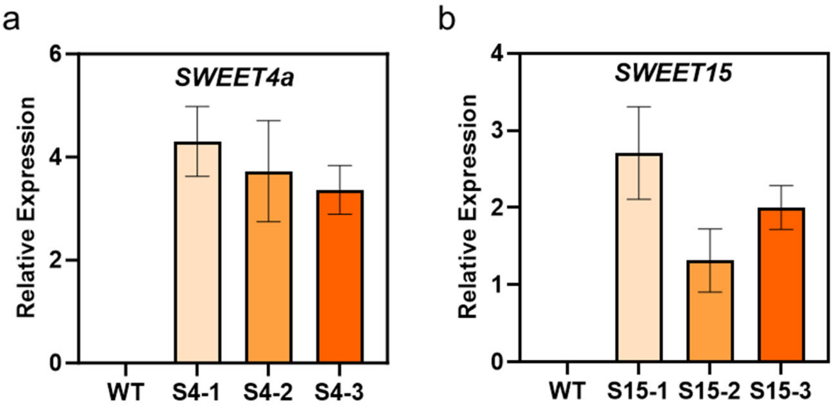

Figure S6. (a, b) Ectopic expression of *NnSWEET4a* and *NnSWEET15* in *Arabidopsis*. Arabidopsis plants harboring 35S::*NnSWEET4a*-eGFP (S4-1, S4-2, S4-3) or 35S::*NnSWEET15*-eGFP (S15-1, S15-2, S15-3) were sampled to determine the relative expression of *NnSWEET4a* (a) and *NnSWEET15* (b). *AtActin2* was used as an internal control. Data are means ± SD, n = 3 for independent biological replicates.

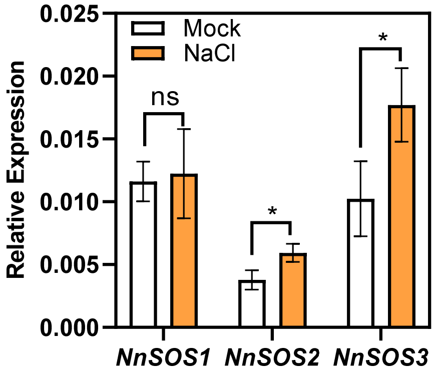

Figure S7. Expression of putative *NnSOS1*, *NnSOS2* and *NnSOS3* in lotus roots under 50 mM NaCl treatment for 24 h detected by qRT-PCR. *NnActin* was used as an internal control. Data are mean ± SD, n = 3 for independent biological replicates, Student's *t*-test, *$P < 0.05$.
